# Antioxidant, Hypotensive, and Antidiabetic Breakthroughs: Bromelain Hydrolysis Unlocks Quinoa’s Peptide Potential - *In Silico* and *In Vitro* Approach

**DOI:** 10.1021/acs.jafc.5c03789

**Published:** 2025-08-25

**Authors:** Maria Lilibeth Manzanilla-Valdez, Sarita Montaño, Cristina Martinez-Villaluenga, Fernanda Zúñiga, Christine Boesch, Alan Javier Hernandez-Alvarez

**Affiliations:** † School of Food Science and Nutrition, 4468 University of Leeds, Leeds LS2 9JT, UK; ‡ Laboratorio de Bioinformática y Simulación Molecular, Facultad de Ciencias Químico Biológicas, Universidad Autónoma de Sinaloa, Culiacan, Sinaloa 80030, México; § Department of Technological Processes and Biotechnology, Institute of Food Science, 593682 Technology and Nutrition (ICTAN-CSIC), Jose Antonio Novais 6, Madrid 28040, Spain; ∥ National Alternative Protein Innovation Centre (NAPIC), Leeds LS2 9JT, UK

**Keywords:** quinoa, bromelain, bioinformatics, 11S globulin, metabolic disorders, molecular dynamics

## Abstract

This study integrates bioinformatics and experimental approaches to characterize bioactive peptides derived from quinoa 11S-globulin (*Chenopodium quinoa* Willd) hydrolyzed*in silico* by stem bromelain (EC3.4.22.32). A total of 109 peptides were generated, of which 14 sequences with more than five amino acids were selected based on molecular docking and dynamics simulations against key metabolic targets (ACE-I, DPP-IV, α-glucosidase, and lipoxygenase). NIYQIS and QDQHQKIR demonstrated the highest binding affinities and hydrogen-bonding interactions, with ADMET predictions confirming their non-toxic and bioavailable profiles. *In vitro*, NIYQIS showed the strongest inhibitory activity against ACE-I (53%), DPP-IV (16.36%) and exhibited the highest antioxidant capacity (ORAC: 0.75 μM TE/μM peptide). Conversely, QDQHQKIR demonstrated the highest α-amylase inhibition (18.43%) and Cu^2+^ chelation (40.4%), supporting its role in carbohydrate metabolism and metal-ion homeostasis. Overall, NIYQIS emerged as the most promising peptide, highlighting the potential of quinoa-derived peptides as functional ingredients to mitigate oxidative stress and metabolic disorders.

## Graphical abstract

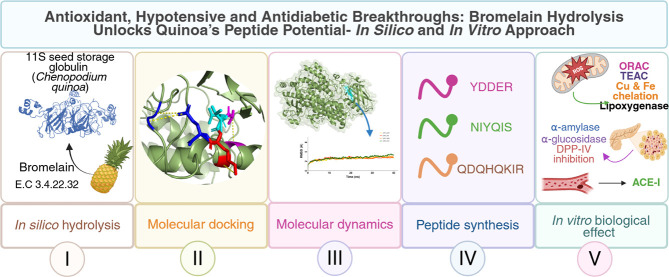

## 1 Introduction

In recent years, protein hydrolysates and peptides have gained significant interest as pharmaceutical agents for chronic diseases, as well as for their ability to enhance food and protein quality.^1^ Protein hydrolysis can be carried out by different processes: enzymatic, chemical, or computational (*in silico*) approaches.^2^ Among these, enzymatic hydrolysis has many advantages, as it avoids extreme conditions while preserving the nutritional value of proteins.^2,3^ On the other hand, complete hydrolysis is rarely achieved, as most enzymes require cofactors for optimal performance.^2,3^ Alternatively, *in silico* bioinformatics tools have emerged as a promising approach for protein hydrolysis, as many proteins have been elucidated and crystallized, allowing for the simulation of enzymatic hydrolysis and prediction of the resulting peptides.^4,5^

A wide range of enzymes have been employed for food protein hydrolysis, such as trypsin, salivary amylase, chymotrypsin, pepsin, papain, bromelain, and ficin, among many others.^6,7^ While gastrointestinal enzymes are the most commonly investigated for identification of bioactive peptides released during the physiological process of digestion,^8^ plant-derived proteases have attracted increasing interest in the food industry.^3^

Plant-based proteases such as papain, stem bromelain, and ficin offer several advantages, including high proteolytic activity, heat-resistance, accessibility, and cost-effectiveness compared to digestive enzymes.^9^ Peptides resulting from protease hydrolysis have demonstrated positive biological effects such as antidiabetic, antioxidant, hypotensive, anticancer, antimicrobial, and hypolipidemic.^8,10–13^ In this context, stem bromelain is a promising enzyme for producing bioactive peptides.^14–16^ Stem bromelain (EC 3.4.22.32) is a cysteine endoprotease present in the pineapple stem, with a molecular weight (MW) of 23.8–37.0 kDa.^17^ Additionally, stem bromelain is a glycoprotein with a single polypeptide and a single carbohydrate side chain.^16^ It exhibits specificity for certain amino acid residues such as Gly, Arg, and Lys.^18^ Stem bromelain has an optimal activity temperature between 50-60 °C, and a wide pH range of 3.9–8.0, with maximum effectiveness at pH 7.1.^15,16,18^ Interestingly, stem bromelain can hydrolyze structural proteins such as collagen, fibrin, and elastin.^16^ Different studies reported its potential in protein hydrolysis to produce peptides,^8,10–13^ with health-promoting properties such as cardiovascular protection, anticancer activity, antidiabetic, and antioxidant properties.^3,16^

Despite the extensive research on bromelain or stem bromelain hydrolysis of common protein substrates, such as casein, soy, wheat, or animal-based proteins, limited attention has been given to its application to pseudocereals like quinoa.^6,15,19^ Quinoa (*Chenopodium quinoa* Willd) is a pseudocereal known for its high protein content compared to cereals, and low or no presence of gliadins (<10%).^20,21^ Quinoa has a protein content of around 15–23 g/100 g, where the major proteins are globulins (37% of total proteins) and albumins (35% of total proteins).^22,23^ Additionally, 11S seed storage globulin (11S-G), also known as “chenopodin”, is the most abundant protein among globulins in quinoa seeds.^22,24^ The 11S-G is a hexamer with six pairs of acidic and basic subunits, connected by disulfide bonds, making it an ideal option for hydrolysis and peptide production.^23^

Bioinformatics tools offer a cost-effective alternative to the conventional experimental approaches by enabling the identification of bioactive peptides without the need for chemical synthesis.^25^ Molecular docking (MD) is employed to discover novel molecules (peptides) that bind and interact with proteins.^25,26^ Extensive literature has shown that MD can predict binding pose, energetically favorable geometry, binding energy (Δ*G*), and interacting residues. Although MD is less accurate than experimental methods,^25–28^ correlating *in silico* predictions with *in vitro* assays enhances its reliability. Different studies have successfully applied MD to assess peptide bioactivity, then synthesized and analyzed them with *in vitro* experiments.^29–32^ To date, limited research has investigated the hydrolysis of quinoa 11S-G by using stem bromelain, through *in silico* and *in vivo* analysis.^29^ Moreover, there are scarce studies evaluating the multifunctional bioactivity of chemically synthesized peptides, especially those derived from stem bromelain hydrolysis.^14–16^

Therefore, the first aim of this study was to hydrolyze the major storage protein of quinoa (11S seed storage globulin, accession code: AAS67036.1) using stem bromelain (EC 3.4.22.32) by *in silico* means to identify potential bioactive peptides. The second aim was to characterize the resulting peptides with different bioinformatics tools, such as molecular docking, molecular dynamics, and ADMET analysis, to predict their bioactivity and pharmacokinetics. Finally, the last objective was to chemically synthesize the selected peptides and evaluate their *in vitro* antidiabetic activities (α-amylase, α-glucosidase, and dipeptidyl peptidase-IV (DPP-IV)), their hypotensive effect (angiotensin I converting enzyme, ACE-I), as well as their antioxidant capacities (oxygen radical absorbance capacity (ORAC), lipoxygenase inhibition (LOX), TROLOX equivalent antioxidant capacity (TEAC), Cu^2+^, and Fe^2+^ chelation).

## 2 Materials and Methods

### 2.1 Reagents

Antidiabetic and antioxidant reagents and enzymes, including DPP-IV, 3,5-dinitrosalicylic acid (DNS), porcine pancreatic α-amylase (PPA), 2-chloro-4-nitro-protocatechuic acid (CNPG3), acarbose, rat intestinal acetone, α-glucosidase, glucose oxidase/peroxidase (GOPOD), Diprotin A (Ile-Pro-Ile), H-Gly-Pro-pNA, ORAC, TROLOX, fluorescein, 2,2’-azobis­(2-amidinopropane)­dihydrochloride (AAPH), 2,2’-azino-bis­(3-ethylbenzthiazoline-6-sulfonic acid (ABTS), and potassium persulfate, were purchased from Sigma-Aldrich (Dorset, UK). Lipoxygenase inhibitor screening assay kit (no. 760700) was purchased from Cayman Chemicals (Ann Arbor, MI, USA). Ethanol and methanol, all HPLC grade, and angiotensin I converting enzyme activity assay kit (CS0002) were obtained from Merck (Darmstadt, Germany). Synthetic peptides (≥ 95% purity) were custom-synthesized and supplied by GenScript Biotech Ltd. (Oxford, UK).

### 2.2 Data Set Creation

Quinoa (*Chenopodium quinoa* Willd) protein sequence was retrieved from UniProt (https://www.uniprot.org/), with the following accession code **AAS67037.1** from 11S-G (*Chenopodium quinoa*).

### 2.3 *In Silico* Hydrolysis and Peptide Characterization

Simulated hydrolysis of quinoa fast adaptive shrinkage threshold algorithm (FASTA) sequence was carried out using the BIOPEP online server (https://biochemia.uwm.edu.pl/biopep/proteins_data_page1.php). Then, the selected sequence (**AAS67037.1**) was input into the “Enzymatic Action” tool, where the stem bromelain enzyme (EC 3.4.22.32) was selected for hydrolysis. The predicted theoretical peptide sequences were submitted to the “Active Fragments Search” tool to assess antidiabetic and antioxidant activity.^33^ Afterward, peptide sequences longer than five amino acids were submitted to PeptideRanker (http://distilldeep.ucd.ie/PeptideRanker/) for bioactivity prediction. Then, peptide sequences were analyzed by ToxinPred (http://crdd.osdd.net/raghava/toxinpred/), selecting “Batch Submission”. A list of toxicological and physicochemical parameters was created, such as toxic prediction, molecular weight (MW), charge, and support vector machine (SVM). Finally, acidic, basic, neutral, and hydrophobic percentages were calculated using the Peptide 2.0 server (https://www.peptide2.com/main_about.php).

### 2.4 Absorption, Distribution, Metabolism, Excretion, and Toxicity (ADMET)

First, quinoa peptide sequences were converted into SMILES (simplified molecular input line entry system) format using the BIOPEP online server (https://biochemia.uwm.edu.pl/biopep/rec_pro1.php?x=43&y=4). Then, SMILES sequences were analyzed using ADMETlab 3.0 online tool (http://admetmesh.scbdd.com/), where carcinogenic potential, hepatotoxicity, and acute oral toxicity were predicted.^34^

### 2.5 Molecular Docking (MD)

All 3D crystal structures were retrieved from PDB (https://www.rcsb.org/): DPP-IV (PDB: 2P8S, 2.20 Å), α-amylase (PDB: 4W93, 1.35 Å), α-glucosidase (PDB: 3W37, 1.70 Å), insulin receptor (INSR) (PDB: 1IR3, 1.90 Å), angiotensin-converting enzyme (ACE) (PDB: 108A, 2.00 Å), and lipoxygenase (PDB: 1N8Q, 2.10 Å). Moreover, all water molecules, ligands, and cations were removed from the PDB files. Finally, all 3D structures used for docking lacked mutations. Molecular docking between proteins (molecular targets) and ligands (quinoa peptides) was predicted using the PepBDB server (http://huanglab.phys.hust.edu.cn/pepbdb/), PyMol 3.0 (https://pymol.org), and UCSF Chimera (https://www.cgl.ucsf.edu/chimera/). Furthermore, for final visualization, the PDBsum online server (https://www.ebi.ac.uk/thornton-srv/databases/pdbsum/) was used. Finally, the complexes that exhibited the highest negative energy were selected for further analysis.

### 2.6 Molecular Dynamics Simulation (MDS) and Trajectory Analysis

For molecular dynamics simulations (MDS), 3D structures were prepared with VMD version 9.3 software. All the MDS systems were carried out using NAMD 2.8,^35^ using GPU-CUDA with NVIDIA Tesla C2070/Tesla C2075 graphics cards. The CHARMM2 and CHARMM27 force fields were used to create the topologies for proteins and lipids, respectively,^36^ while the TIP3 model was used for water molecules. The system was solvated using the *psfgen* software in the VMD program.^37^ All systems were minimized for 1000 steps, followed by equilibrium under constant temperature and pressure conditions (NPT) for 1 ns with protein and lipid atoms restrained. Subsequently, 40 ns of MDS was run, considering all proteins as soluble, without position restraints under PBC (periodic boundary conditions) and using an (Isothermal–Isobaric) NPT ensemble at 310 K. The MDS were performed in the Laboratory of Molecular Modeling and Bioinformatics at the Facultad de Ciencias Químico Biológicas, Universidad Autónoma de Sinaloa, and in the Hybrid Cluster Xiuhcoatl (http://clusterhibrido.cinvestav.mx) of the CINVESTAV-IPN, México. The stability and conformational changes of the system were evaluated by analyzing root mean square deviation (RMSD), root mean square fluctuation (RMSF), and the radius of gyration (Rg). Trajectory analysis was calculated with the Carma software.^38^ Molecular graphics were performed in the R studio. Finally, peptides showing the best outcomes after MD were selected for synthesis and *in vitro* biological evaluation.

### 2.7 Peptide Synthesis

Selected synthetic peptides YDDER, NIYQIS, and QDQHQKIR were custom-synthesized by GenScript Biotech Ltd. (Oxford, United Kingdom), with a purity of 95%. Furthermore, the synthetic peptides were tested for solubility, and the most suitable solvent was reported. Finally, YDDER and QDQHQKIR were dissolved in Milli-Q-Water, while NIYQIS was dissolved in DMSO. Peptide concentration was calculated by mass (g) divided by molecular weight (g/mol) per unit of volume expressed in L.

### 2.8 *In Vitro* Antidiabetic Assay

#### 2.8.1 α-Amylase Inhibition

α-Amylase inhibition activity was performed using the 3,5-dinitrosalicylic acid (DNS) assay according to Zulfiqar et al.^39^ Briefly, 100 μL of sample (synthetic peptides, 100–500 mM) or positive control (acarbose) was added to 100 μL of porcine pancreatic α-amylase (PPA) solution and incubated for 10 min at 37 °C. Then, 50 μL of substrate (2-chloro-4-nitro-protocatechuic acid (CNPG3) (2 mM)) was added per well. Finally, absorbance was recorded at 405 nm (SPARK-10M, TECAN, Switzerland) at 37 °C for 10 min in 1-min intervals. The percentage of enzyme inhibition was calculated in relation to 100% enzymatic activity in the negative control (phosphate-buffered saline (PBS) buffer, enzyme, and substrate).

#### 2.8.2 α-Glucosidase Inhibition

α-Glucosidase inhibition assay was measured following the method of Vilcacundo et al., with slightly modifications.^40^ First, 100 μL of sample (synthetic peptides, 100–500 mM), positive control (1 mM acarbose), and/or negative control (Milli-Q-water), were added to 50 μL of rat intestinal acetone α-glucosidase (1 U/mL in 0.1 M, maleate buffer pH 6.9), and the mixture was incubated at 37 °C for 5 min. Then, 50 μL of substrate (2 mM maltose) was added into each tube, which was performed in a Thermomixer (37 °C, 1000 rpm, for 30 min). Finally, the reaction was stopped at 100 °C for 5 min. The glucose concentration in the reaction was measured using GOPOD (glucose oxidase/peroxidase) from Megazyme, with absorbance measured at 560 nm (Gen5 software, version 1.1, BioTek Instruments, Winooski, VT, USA). The percentage of inhibition was calculated relative to the non-inhibited control (negative control).

#### 2.8.3 Dipeptidyl Peptidase IV Inhibitory Activity

Dipeptidyl peptidase-IV (DPP-IV) inhibitory activity was measured following the method by Vilcacundo et al., with slightly modifications.^40^ Briefly, 30 μL of human DPP-IV (0.26 mU per test well) was added to all wells, followed by 20 μL of sample (synthetic peptides, 125 – 500 mM) or positive control (Diprotin A, 3 μg). For the negative control 70 μL of Tris Buffer was added. Finally, 100 μL of substrate (H-Gly-Pro-pNA, 100 mM) was added to all wells. The reaction was performed at 37 °C in the microplate reader (BioTek Instruments, Winooski, VT, USA), and the reaction was read at 405 nm for 30 min, with reading intervals of 2 min. Finally, data were plotted and fitted to a logarithmic regression to obtain dose–response curves.

### 2.9 *In Vitro* Antioxidant Assay

#### 2.9.1 Oxygen Radical Absorbance Capacity Assay

Oxygen radical absorbance capacity (ORAC) assay was performed following the method of Carvalho-Oliveira et al.^41^ Briefly, 30 μL of peptide (different concentrations 50–1000 μM) or standard (Trolox at 0–160 μM) were placed in a black 96-well plate. The reaction mixture comprised 180 μL of fluorescein (116.9 nM) and 90 μL of 2,2’-azobis­(2-amidinopropane) dihydrochloride (AAPH) (40 mM). Finally, the fluorescence was recorded every 2 min for 150 min at excitation and emission wavelengths of 485 and 520 nm, respectively. All reactions were performed at 37 °C. The results were expressed as μmol Trolox equivalent (TE)/μmol of peptide. Quantification and interpretation of the data were done by Gen5 software, version 1.1 (BioTek Instruments, Winooski, VT, USA).

#### 2.9.2 Trolox Equivalent Antioxidant Capacity Assay

The Trolox equivalent antioxidant capacity (TEAC) analysis was performed as described by Sanchez-Velazquez et al.^42^ In order to produce ABTS (2,2’-azino-bis­(3-ethylbenzthiazoline-6-sulfonic acid) radical cation (ABTS^**●+**^), ABTS (7 mM) were mixed with potassium persulfate (2.45 mM) in the dark at room temperature (RT 18–21 °C) for 16 h. Then, 20 μL of sample (synthetic peptides, 100–500 mM), standard (Trolox, 1000–50 μM), and positive control (BHT, 500 μM) were added to a 96-well plate, followed by the addition of 200 μL of ABTS^**●+**^ solution. Finally, the reaction was measured at 0 and 6 min at 734 nm (SPARK-10M, TECAN, Switzerland). The percentage of inhibition was calculated as follows:

$$\%\mathrm{TEAC}\;\mathrm{inhibition}=\frac{\mathrm{Abs sample}0\min-\mathrm{Abs sample}6\min}{\mathrm{Abs sample}0\min-\left(\frac{\mathrm{Abs blank}0\min-\mathrm{Abs blank}\;6\min}{\mathrm{Abs blank}0\min}\right)}\times 100$$

#### 2.9.3 Copper (Cu^2+^) and Iron (Fe^2+^) Chelation

Cu^2+^ chelation assay was determined according to Sanchez-Velazquez et al., with slight modifications.^42^ Briefly, 21 μL of sample (synthetic peptides, 7.8–500 mM) or positive control (EDTA, 0.1 μg/μL) was added to 185 μL of buffer (sodium acetate, 50 mM, pH 6.0), and then 15 μL of copper solution (10 μg) and 9 μL of pyrocatechol violet (2 mM) were added for the reaction. For the blank well, 205 μL of buffer was added to the copper and pyrocatechol violet reaction mixture. The reaction was incubated for 10 min at 37 °C and then read at 632 nm (SPARK-10M, TECAN, Switzerland). Cu^2+^ chelating activity was calculated as:

$$\%\mathrm{Chelating}\mathrm{activity}=\frac{\mathrm{Abs}\;\mathrm{control}-\mathrm{Abs}\;\mathrm{sample}}{\mathrm{Abs}\mathrm{control}}\times 100$$

Fe^2+^ chelation assay was measured according to Sanchez-Velazquez et al. with slightly modifications.^42^ 25 μL of sample (synthetic peptides, 7.8–500 mM) or positive control (EDTA, 0.1 μg/μL) was added to 225 μL of buffer (sodium acetate, 100 mM, pH 4.9), then 15 μL of iron solution (0.1%, w/v) and 9 μL of ferrozine (40 mM) were added to start the reaction. For the blank well, 250 μL of buffer was added to the iron solution and ferrozine. The reaction was incubated 10 min at 37 °C and then read at 562 nm (SPARK-10M, TECAN, Switzerland). Fe^2+^ chelating activity was calculated as described previously for copper.

#### 2.9.4 Lipoxygenase Inhibitor Screening Assay

Lipoxygenase inhibitory activity assay (LOX) was determined following the manufacturer’s instructions (#760700).^43^ Thus, peptide concentrations (31.25–125 μM) were prepared with the assay buffer and tested in triplicate. First, 10 μL of the peptides at each concentration was mixed with 90 μL of 15-LOX enzyme (soybean lipoxygenase) in a 96-well plate. Then, three wells each contained assay buffer as blank (without enzyme), assay buffer as vehicle (with enzyme), and 15-LOX as positive control. Afterward, 10 μL of linoleic acid (1 mM) (substrate) was added to all wells and shaken for 10 min at RT. Finally, 100 μL of chromogen was added to all wells, and the plate was sealed and shaken for an additional 5 min at RT. The final reaction was measured at 490 nm, and the percentage of inhibition was calculated as.

$$\%\mathrm{inhibition}=\left[1-\frac{\mathrm{Corrected}\mathrm{values}\mathrm{of}\mathrm{peptide}}{\mathrm{Corrected}\mathrm{values}\mathrm{of}\mathrm{vehicle}}\right]\times 100$$

### 2.10 Angiotensin I Converting Enzyme Inhibitory Activity

ACE-I inhibitory assay was determined by ACE-I activity assay kit (fluorometric, CS0002).^44^ This assay is based on the hydrolysis of angiotensin I by ACE to yield angiotensin II. Briefly, all reagents were diluted in the assay buffer, and a total of 50 μL of peptide sample, positive control (captopril PHR1307), and assay buffer (blank) were added to a 96-well black plate. The standard was added at 0, 0.1, 0.2, 0.3, 0.4, 0.5, 0.6, and 0.8 nmol and adjusted to a final volume of 100 μL. Then, 50 μL of substrate was added only to peptide samples (125 – 500 μM), positive control (50 μM), and blank. Finally, fluorescence (λex, 320 nm; λem, 405 nm) was measured in five cycles for 5 min. Linear regressions were determined to calculate the enzymatic activity for each sample per concentration. Thus, one unit was defined as the amount of enzyme that releases 1 nmol of fluorescent substrate in 1 min at 37 °C. Finally, the inhibition (%) of each peptide and positive control was determined using regression analysis of the corresponding dose–response curves.

### 2.11 Statistical Analysis

All analyses were processed using GraphPad Prism version 10.0.0 for Windows (GraphPad Software, Boston, Massachusetts, USA) and Minitab, LLC (2025). Data were evaluated using one-way ANOVA (*p* < 0.05), followed by Tukey posthoc test. All results are presented as mean ± standard deviation, with all analyses conducted in quintuplicate.

## 3 Results and Discussion

### 3.1 *In Silico* Hydrolysis and Peptide Characterization

Several studies have reported different accession codes for 11S-G, raising the question of which structure is the most suitable for research applications.^29,45,46^ Thus, a comprehensive literature review was performed to catalog all available 11S-G FASTA sequences. Then, a BLAST (basic local alignment search tool) was performed to assess the homology between sequences. Finally, 11S-G (AAS67037.1) was selected as an ideal option for *in silico* hydrolysis using stem bromelain (EC 3.4.22.32).

This simulation generated 109 peptide fragments (Figure S1), of which 95 were fragments with less than four amino acids, mostly dipeptides, whereas 14 of the peptides were more than five amino acids in length.

According to the International Union of Pure and Applied Chemistry (IUPAC), peptides consist of at least 2–20 amino acids.^47,48^ Furthermore, short and ultrashort peptides have been described as sequences with less than 10 and 7 amino acids, respectively,^48^ and have been reported to offer many advantages, such as low allergenicity, low or no toxicity, easy recognition by the molecular target, ability to penetrate the cell membrane and the blood–brain barrier, easy availability, ease of design, and cost-effectiveness.^47^ However, dipeptides, tripeptides, and tetrapeptides are less specific sequences; moreover, they could bind and interact with several parts of the molecular target.^26,49^ Therefore, peptides with a length between five and 20 amino acids were selected for further *in silico* analysis.

Peptide characterization is an important feature in the development of functional ingredients/foods, and nutraceuticals.^10^ One of the critical parameters to evaluate is toxicity, as therapeutic peptides must be non-toxic and should not affect the cell metabolism to be considered for synthesis.^4,50^ To assess these parameters, all quinoa peptides produced from stem bromelain hydrolysis, with more than five amino acids (14 in total), were analyzed by ToxinPred, Peptide2.0, and PeptideRanker. The results from quinoa peptide characterization are depicted in Table 1.

**Table 1:**
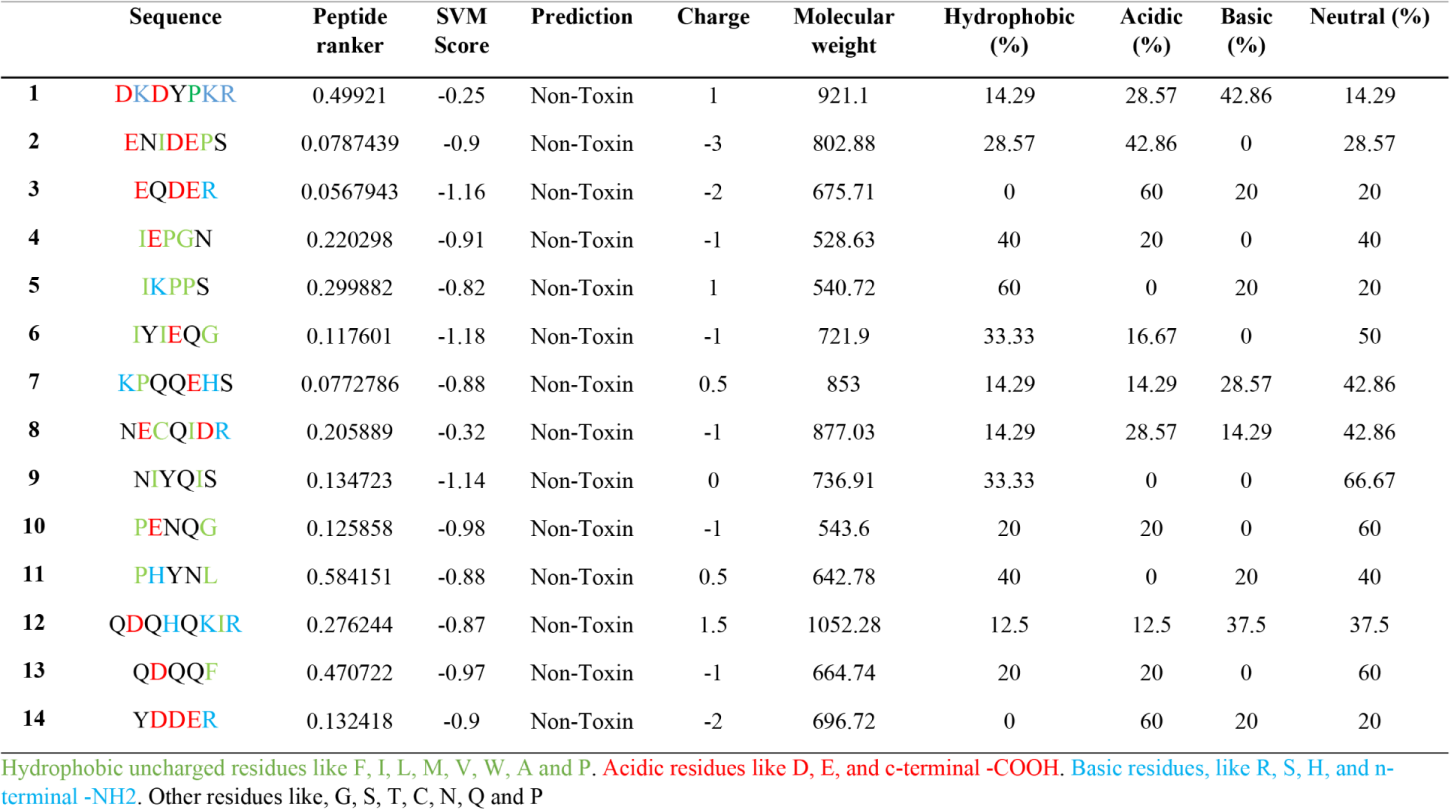
Peptides ≥ 5 Amino Acids Released from Stem Bromelain Hydrolysis of 11S Seed Storage Globulin *Chenopodium Quinoa* (AAS67037.1)

PeptideRanker was used to predict the theoretical bioactivity of quinoa peptides, yielding a score from 0 to 1, with 1 representing a highly bioactive peptide.^51^ The highest scores were presented by PHYNL (0.58), DKDYPKR (0.49), QDQQF (0.47), IKPPS (0.30), and QDQHQKIR (0.28). On the other hand, ENIDEPS (0.08), KPQQEHS (0.08), and EQDER (0.06) showed the lowest scores. Even though PeptideRanker is an important tool for bioactivity prediction, it has limitations, as it does not provide a precise measurement of specific biological activities.^50^ Hence, *in vitro* and *in vivo* validation using synthetic peptides remains essential.^52^

Furthermore, quinoa peptides were analyzed to predict molecular weight, charge, hydrophobicity, and acidic, basic, and neutral characteristics (Table 1). The molecular weight ranged between 528.63 and 1052 kDa, with a net charge varying between −3.0 and 1.5, mostly exhibiting a negative charge. Peptide solubility can be correlated with the net charge, as positively charged peptides can be considered basic, negatively charged peptides are considered acidic, while a zero net charge is considered neutral.^53^ It can be observed that most quinoa peptides are negatively charged, and only one peptide (NIYQIS) has a zero net charge. Regarding solubility characteristics, 38.77% of quinoa peptides generated through simulated hydrolysis had neutral properties: NIYQIS (66.7%), PENQG (60%), IYIEQG (50%), KPQQEHS (42.7%), NECQIDR (42.7%), and IEPGN (40%). Only two peptides were mostly basic: DKDYPKR (42.9%) and QDQHKIR (37.5%). The toxin analysis predicted that all quinoa peptides were nontoxic. Computational methods that predict toxicity are cost-effective, reduce time (synthesis), and facilitate peptide design.^4^ A comprehensive literature review to catalog all peptides reported to exhibit antidiabetic activity *in vitro* or *in vivo* was conducted. These candidate sequences were then scored using PeptideRanker; however, it was found that this tool exhibited limited sensitivity for antidiabetic peptide identification. Examination of the active peptides revealed a common motif: hydrophobic or neutral amino acids positioned at the N- or C-terminus conferred potent inhibition of α-amylase, α-glucosidase, and DPP-IV and hypotensive activity. Consequently, peptides exhibiting these terminal characteristics (IEPGN, PENQG, QDQQF, YDDER, NIYQIS, and QDQHQKIR) were prioritized for the subsequent *in silico* ADMET profiling, MD, and MDS. These combined criteria ensured that only the most promising antidiabetic and hypotensive candidates advanced to detailed computational evaluation.

### 3.2 ADMET

Pharmacokinetic properties are crucial in the development and synthesis of bioactive peptides.^34^ Thus, absorption, distribution, metabolism, excretion, and toxicity (ADMET) are essential to evaluate the drug-likeness potential of each compound and assess possible side effects.^5^ Although in a previous section ToxinPred was used, this tool was developed to predict and design toxic or nontoxic peptides using machine learning (SVM), BLAST, and MERCI techniques. Furthermore, this online tool predicts the overall behavior of the peptide; it does not provide detailed information about pharmacodynamics. On the other hand, ADMET3.0 is a platform for evaluating ADMET-related parameters as well as physicochemical properties and medicinal chemistry, all of which are involved in the drug discovery process. Thus, ADMET3.0 is a more in-depth tool for assessing possible toxicity in different organs—especially the liver (metabolism) and kidneys (excretion).^5,28,34,54^ Therefore, ADMETlab 3.0 server (https://admetmesh.scbdd.com/) was used to evaluate the absorption, distribution, metabolism, excretion, drug-induced toxicity, and medicinal chemistry of the quinoa-derived peptides IEPGN, PENQG, QDQQF, YDDER, NIYQIS, and QDQHQKIR (Table 2). Caco-2 permeability results indicated a consistent trend, with all the quinoa peptides exhibiting low permeability (<−5.84). Conversely, human intestinal absorption (HIA) prediction varied among peptides: QDQQF has a high probability of being absorbed, while IEPGN, YDDER, and QDQHQKIR showed low probability. This is in agreement with the literature, as compounds or drugs with a MW less than 500 kDa showed low gastrointestinal absorption.^55^ Furthermore, all six quinoa peptides revealed excellent blood–brain barrier penetration (BBB) and plasma protein binding (PPB) capacity. In this regard, NIYQIS (34.4%) and IEPGN (33.4%) exhibited the highest PPB percentage. Thus, PPB and BBB penetration are key factors associated with biodistribution efficiency and safety to specific target tissues.^56^

**Table 2:**
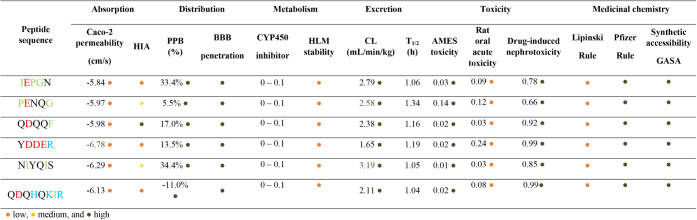
Quinoa-Derived Peptides Absorption, Distribution, Metabolism, and Excretion Analysis^a^

a Optimal conditions: Caco-2 permeability higher than −5.15 cm/s. PPB < 90%. CYP450 < 1.0 is considered an inhibitor. CL plasma >15 mL/min/kg is high clearance, 5–15 mL/min/kg is moderate clearance, and <5 mL/min/kg is low clearance. T1/2 short life: 2–4 h. Rat oral acute toxicity: > 500 mg/kg is low toxicity, and <500 mg/kg is high toxicity. GASA: easy and high to synthetize. AMES; BBB: blood-brain barrier penetration, CL: plasma clearance, CYP: cytochrome 450, HIA: human intestinal absorption, HLM: human liver microsomal, PPB: plasma protein binding

Drug metabolism is another important parameter to measure, especially cytochrome P450 (CYP450).^57^ Cytochromes are widely expressed in human tissues, mostly synthesized by intestinal cells and hepatic tissue.^57^ Moreover, CYP450 enzymes metabolize a wide range of drugs and compounds.^57^ In this study, all six quinoa peptides presented CYP450 inhibition values between 0 and 0.1 (Table 2). Furthermore, the human liver microsomal (HLM) score revealed that quinoa peptides have low stability in liver metabolism. Compounds metabolized rapidly by HLM exhibit lower bioavailability.^58,59^ Additionally, HLM is used to determine the effect of chemical inhibitors on human metabolism.^59^ In general, peptides are metabolized by two pathways: endocytosis followed by lysosomal degradation or hydrolysis by brush border enzymes before tubular absorption.^60^

Excretion results showed that IEPGN, PENQG, QDQQF, YDDER, NIYQIS, and QDQHQKIR peptides had an excellent plasma clearance (CL) value, which ranged between 1.65 and 3.19 mL/min/kg. Peptides with a CL < 5 mL/min/kg are considered to have efficient excretion. In addition, the half-life of the quinoa peptides ranged from 1.04 to 1.34 h, which is an acceptable range for drug candidates, as compounds with an excretion half-life of less than 4 h are typically considered suitable for pharmaceutical applications.^60^ In general, peptides are mainly excreted through the kidneys by glomerular filtration.^56,60^

Rat oral acute toxicity showed that the peptides IEPGN, PENQG, QDQQF, YDDER, NIYQIS, and QDQHQKIR (Table 2) presented a low probability of being toxic. Conversely, drug-induced nephrotoxicity revealed that quinoa peptides might have a high probability of being toxic to the kidneys. However, the prediction for excretion in this online tool showed acceptable parameters for renal filtration. Hence, experimental validation of *in silico* analysis, using *in vitro* cell culture and *in vivo* models, is essential to confirm these findings.^24,61^

Finally, the quinoa-derived peptides did not comply with Lipinski’s rule, although they met the criteria of the Pfizer rule. Lipinski’s rule consists of four criteria: no more than five hydrogen bond donors, no more than 10 hydrogen bond acceptors, a molecular mass less than 500 Da, and a Clog *p* value < 5 to ensure the compound is not too lipophilic.^62–64^ The Pfizer rule, an empirical filter used in medicinal chemistry, states that compounds with a ClogP < 3 and a total polar surface area (TPSA) >75 Å^2^ are less likely to exhibit off-target toxicity. Compounds exceeding these thresholds have been reported to be approximately 2.5 times more likely to show toxicity.^62–64^ Therefore, the quinoa peptides exceeded the molecular mass threshold, resulting in noncompliance with Lipinski’s rule.

Overall, the synthetic accessibility GASA parameter revealed that IEPGN, PENQG, QDQQF, YDDER, NIYQIS, and QDQHQKIR will be easy to synthesize; this could be related to the MW and amino acid sequence of quinoa peptides.

The selected quinoa-derived peptides exhibit promising bioactive properties, particularly due to their systemic distribution and ability to cross the blood–brain barrier. However, their low intestinal absorption and rapid hepatic metabolism may limit their effectiveness when administered orally, underscoring the need to explore alternative formulation strategies or routes of administration. While no general toxicity was observed, the potential risk of nephrotoxicity requires further experimental validation. Future studies should focus on *in vitro* and *in vivo* assessments to confirm the peptides’ bioactivity and investigate approaches for improving their stability and bioavailability in functional foods and nutraceutical applications. The next steps in research should include validation of absorption and renal safety using *in vitro* and *in vivo* models, evaluation of formulation strategies, such as encapsulation or structural modifications to improve oral bioavailability, and conducting molecular interaction studies using MD and MDS to further elucidate their functional potential.

### 3.3 Molecular Docking (MD)

In general, MD is widely used to predict interactions between protein targets and small molecules (drugs, compounds, or peptides).^25^ This technique estimates the binding pose—the most energetically favorable geometry during binding—and presents the results in a three-dimensional (3D) structure.^25,27^ It has been demonstrated that MD is a reliable and essential tool for drug or nutraceutical discovery and screening.^62,65^

Therapeutic peptides may play a critical role in the management and treatment of chronic diseases such as cardiovascular conditions, hypotension, obesity, or diabetes mellitus.^8,66,67^ Diabetes mellitus, particularly diabetes mellitus type 2 (T2DM), is a multifactorial syndrome mainly characterized by hyperglycemia.^8,68^ Studies have reported that hypoglycemic peptides are directly involved in stimulating insulin secretion, inhibiting DPP-IV, and suppressing carbohydrate-hydrolyzing enzymes such as α-amylase and α-glucosidase.^8,11,69^

Given these considerations, this study aims to evaluate the interactions between six quinoa peptides (IEPGN, PENQG, QDQQF, YDDER, NIYQIS, and QDQHQKIR) and key proteins related to T2DM and metabolic syndrome, including α-amylase and α-glucosidase (involved in carbohydrate digestion and glucose metabolism), DPP-IV (a regulator of incretin hormones), angiotensin-converting enzyme (ACE-I, implicated in vascular health), insulin receptor (INSR, essential for insulin signaling), and lipoxygenase (linked to inflammatory pathways). The docking simulation of quinoa peptides with these targets and active sites is depicted in Table 3 and Figure 1 (Figure S2). Thus, the conformation with the lowest free energy value for binding (Δ*G* kcal/mol) was selected for the 3D simulation.

**Table 3:** Docking Score, Gibbs Binding Free Energy, and Interacting Residues of Quinoa Peptides: IEPGN, PENQG, QDQQF, YDDER, NIYQIS, and QDQHQKIR with Protein Targets^a^

Peptide sequence	Gibbs free energy (kcal/mol)	Hydrogen bonds	Salt bridge
**α-Amylase PDB: 4W93**			
IEPGN	–131.014	Thr163, Glu233, His299	-
PENQG	–135.088	Thr163	-
QDQQF	–156.774	Asp356	-
YDDER	–138.554	Arg303, His305, Asp356	-
NIYQIS	–192.767	Asp197, His299, Asp356	-
QDQHQKIR	–197.79	Gln63, His299	-
**α-Glucosidase PDB: 3W37**			
IEPGN	–117.468	Arg93, Arg102, Glu105	-
PENQG	–128.765	Tyr243	-
QDQQF	–142.173	Asp232, Arg552	-
YDDER	–143.699	Asp568, Asp630	-
NIYQIS	–172.220	Asn475, Lys506, Asp630	-
QDQHQKIR	–162.185	Asp666, Arg699, Glu756, His786	-
**DPP-IV PDB: 2P8S**			
IEPGN	–115.780	Asp545	-
PENQG	–152.193	Arg125, His740	-
QDQQF	–162.002	Arg125, Lys554, Ser630, Tyr662, Asn710	-
YDDER	–155.951	Arg125, Glu205, Asp545, Val546, Trp627, Trp629	-
NIYQIS	–187.194	Tyr43, Asp47, Tyr48, Asn51, Leu561, Ala564, Trp629, Tyr752	-
QDQHQKIR	–219.367	Asn51, Glu205, Asp545, Tyr547, Trp629, Ser630, Asn710, His740	-
**INSR PDB:1IRK**			
IEPGN	–120.534	His1057, Asp1143, Thr1145, His1268,	-
PENQG	–128.604	Thr1145	-
QDQQF	–145.536	Glu1022, Ala1023, Arg1026, Arg1061, Leu1078	-
YDDER	–126.472	His1058, His1268, Ser1270	-
NIYQIS	–158.215	Gln1111, Thr1145, Ser1270,	-
QDQHQKIR	–170.335	Gln1107, Glu1115, Asp1143, Phe1144, Thr1145, Asp1266, Leu1267	-
**ACE-I PDB: 108A**			
IEPGN	–124.299	Ala356, His383, Glu384, His387	-
PENQG	–144.254	Asn66, Asn70, His353, Lys368, Tyr523	-
QDQQF	–172.671	Asn66, Asn70, Leu139, Ser355, Ala356, Tyr360, His387	-
YDDER	–159.079	Tyr62, Lys118, Asp121, Glu123, Arg124, Ser516, Ser517	-
NIYQIS	–205.069	Tyr51, Asn66, Thr92, Lys118, Asp121, Ser355, Ala356	-
QDQHQKIR	–201.398	Asn66, Asp358, Tyr360, Tyr394, Arg402, Gly404, Arg522	-
**Lipoxygenase PDB: 1N8Q**			
IEPGN	–110.892	Arg378, Arg386, Asp428, Asp431	-
PENQG	–131.979	Asn375, Arg378, Asn521, Val594, Gln598	-
QDQQF	–142.414	Val128, Ser129, Thr131, Asn788	-
YDDER	–144.274	Tyr394, Glu584	-
NIYQIS	–162.574	Thr91, Ala94, Gln96, Ser129, Pro789, Asn790	-
QDQHQKIR	–161.546	Pro435, Tyr436, Arg439, Arg580, Tyr581, Glu584	-

a Ala: alanine, Arg: arginine, Asn: asparagine, Asp: aspartic acid, Cys: cysteine, Glu: glutamic acid, Gln: glutamine, Gly: glycine, His: histidine, Ile: isoleucine, Leu: leucine, Lys: lysine, Met: methionine, Phe: phenylalanine, Pro: proline, Ser: serine, Thr: threonine, Trp: tryptophan, Tyr: tyrosine, and Val: valine.

**Figure 1:**
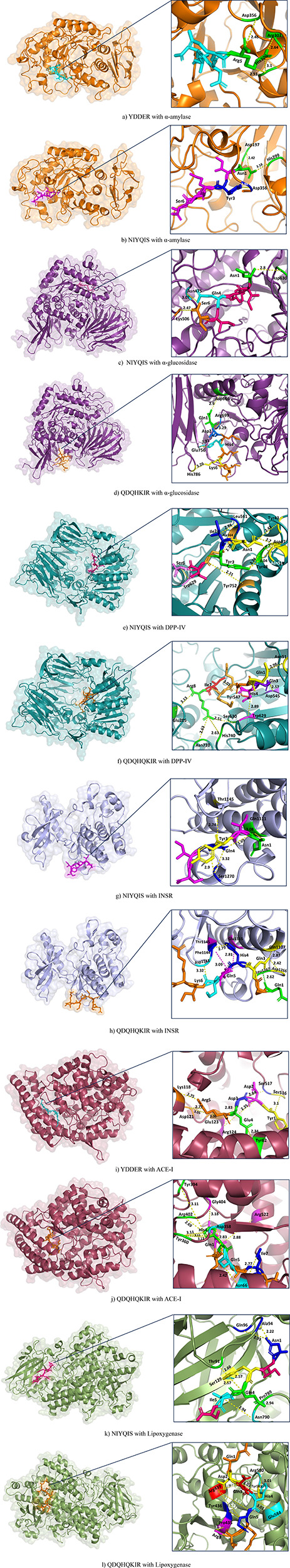
General view of protein–ligand interactions showing the residues from the active site involved in making the interactions with the ligand (quinoa peptides). (a) NIYQIS with α-amylase (PDB), (b) YDDER with α-amylase, (c) NIYQIS with α-glucosidase, (d) QDQHQKIR with α-glucosidase, (e) NIYQIS with DPP-IV, (f) QDQHQKIR with DPP-IV, (g) NIYQIS with INSR, (h) QDQHQKIR with INSR, (i) YDDER with ACE-I, (j) QDQHQKIR with ACE-I, (k) NIYQIS with lipoxygenase, and (l) QDQHQKIR with lipoxygenase.

In general, the results revealed that IEPGN, PENQG, QDQQF, YDDER, NIYQIS, and QDQHQKIR interacted with all of the receptors to varying degrees (Table 3). The inhibition of α-amylase and α-glucosidase plays an important role in reducing glucose in the bloodstream.^70^ These enzymes hydrolyze α-d-(1,4)-glycosidic bonds mainly into oligosaccharides, trisaccharides, maltose, and maltotriose.^71^ Hence, the inhibition of α-amylase and α-glucosidase can delay the absorption of carbohydrates in the intestine, thus reducing glucose spikes.^70,72^

Regarding α-amylase (PDB: 4W93) inhibition, IEPGN presented the highest free energy at −131.014 kcal/mol and formed three hydrogen bonds at Thr163, Glu233, and His299. Furthermore, YDDER (−138.554 kcal/mol) formed three hydrogen bonds at Arg303, His305, and Asp356 (Figure 1a). Interestingly, NIYQIS revealed a free energy of −192.767 kcal/mol with three hydrogen bonds at Asp197, His299, and Asp356 (Figure 1b). The lowest free energy was observed for QDQHQKIR at −197.790 kcal/mol, forming two hydrogen bonds at Gln63 and His299. It can be observed that residues Thr163, Asp356, and His299 are consistently present in quinoa peptides. Montbretin A and caffeic acid are known inhibitors of the α-amylase enzyme and have been reported to bind at the active sites Asp197 and Glu233, respectively.^73^ The binding sites of IEPGN (Glu233) and NIYQIS (Asp197) correspond to the same cavity as these known inhibitors. Furthermore, another blind-docking study revealed the site-specific cavity of acarbose binding to Pro4, Arg252, Trp280, His331, Pro332, Gly403, Pro405, and Arg421, and Diprotin A binding to Trp59, Gln63, Leu162, Ala198, and Ile235.^74^ Notably, in this study, QDQHKIR exhibited a similar binding residue to Diprotin A (Table 3).

In α-glucosidase (PDB: 3W37) inhibition, IEPGN presented three hydrogen bonds at Arg93, Arg102, and Glu105, with a free energy of −117.468 kcal/mol. Interestingly, QDQQF (−142.173 kcal/mol) and YDDER (−143.699 kcal/mol) showed similar free energy values. Additionally, QDQQF (Asp232 and Arg552) and YDDER (Asp568 and Asp630) presented two hydrogen bonds with an affinity to the Asp residue. The lowest energy was presented by NIYQIS (−172.220 kcal/mol), with three hydrogen bonds (Asn470, Lys506, and Asp630) interacting with the molecular target (Figure 1c). Notably, QDQHQKIR showed the highest number of interactions, involving Asp666, Arg699, Gly756, and His786 (Figure 1d), all of which were hydrogen bond. Tagami et al. reported the site-specific cavity of acarbose against α-glucosidase enzyme at Trp329, Asp357, Ile358, Ile396, Asp398, Trp432, Trp467, Phe476, Trp565, Arg552, Asp568, Asp597, Phe601, and His626.^75^ Interestingly, QDQQF (Arg552, H-bonds) and YDDER (Asp568, H-bonds) exhibited the same interaction residues as acarbose in the pocket site of α-glucosidase. Moreover, QDQHKIR and NIYQIS showed several interactions near the binding cavity.

DPP-IV is an enzyme that affects incretin hormones such as glucagon-like peptide-1 (GLP-1) and gastric inhibitory peptide (GIP), which are responsible for glucose homeostasis, insulin secretion, and glucagon regulation.^76–78^ Therefore, DPP-IV inhibition increases the release of incretins (GLP-1 and GIP) in the bloodstream, reducing hyperglycemia in the postprandial phase.^30,77,79^ In this study, the inhibition of DPP-IV (PDB: 2P8S) revealed that YDDER (−152.93 kcal/mol Δ*G*) formed six hydrogen bonds at Arg125, Glu205, Asp545, Val546, Trp627, and Trp629. Furthermore, QDQQF and YDDER shared similarities in MW and negative charge, yet QDQQF is hydrophobic compared to YDDER (Table 1). Notably, NIYQIS (Tyr43, Asp47, Tyr48, Asn51, Leu561, Ala564, Trp629, and Tyr752) and QDQHQKIR (Asn51, Glu205, Asp545, Tyr547, Trp629, Ser630, Asn710, and His740) formed the highest number of interactions, with eight hydrogen bonds each, and exhibited the lowest free energy (Figure 1e,f). Furthermore, NIYQIS and QDQHQKIR had a significant proportion of neutral residues (Table 1), primarily containing Ile (I) and Gln (Q) residues. Several studies have reported the binding sites of known DPP-IV inhibitors—alogliptin and linagliptin (Tyr547 and Trp629), sitagliptin and teneligliptin (Asn710), vildagliptin and saxagliptin (Arg125, Tyr547, Ser630, Val656, Trp659, Tyr662, Tyr666, Asn710, and Val711). It is important to highlight that Glu205, Glu206, and Tyr662 residues play a key role in DPP-IV inhibition as these are located within the enzyme’s active site pocket.^80,81^ Notably, QDQQF (Arg125, Tyr662, and Asn710), YDDER (Arg125, Glu205, and Trp629), and QDQHQKIR (Glu205, Tyr547, Ser630, and Asn710) exhibited interaction patterns within the DPP-IV pocket cavity, similar to those of DPP-IV inhibitors.^80,82^ Thus, QDQQF, YDDER, and QDQHQKIR showed remarkable inhibitory activity against this enzyme, highlighting their potential as antidiabetic peptides.

Insulin activates a wide range of biological processes, through two tyrosine kinase receptors.^83–85^ The INSR activates and initiates a phosphorylation pathway that regulates cellular and glucose metabolism.^84^ In this regard, after carbohydrates are ingested, the pancreas releases insulin, and the INSR recognizes the domain, triggering phosphorylation that activates AKT, leading to GLUT4 translocation and glucose uptake into the mitochondria.^84,86^ In this study, the insulin receptor was retrieved from PDB: 1IRK; thus, IEPGN (−120.534 kcal/mol Δ*G*) exhibited four hydrogen bonds at His1057, Asp1143, Thr1145, and His1268. Notably, QDQQF (−145.536 kcal/mol Δ*G*) formed five hydrogen bond interactions at Glu1022, Ala1023, Arg1026, Arg1061, and Leu1078, while YDDER (His1058, His1268, and Ser1279) and NIYQIS (Gln1111, Thr1145, and Ser1270 – Figure 1g) presented three hydrogen bonds. Interestingly, the free energies differed from each other, as NIYQIS (−158.215 kcal/mol) had the lowest energy compared to YDDER (−126.476 kcal/mol). The free energy, or predicted binding energy, estimates the proximity between the protein and the ligand (peptide), meaning that lower free energy corresponds to a more energetically favorable interaction.^25^ Finally, QDQHQKIR exhibited seven hydrogen bonds at Gln1107, Glu1115, Asp1143, Phe1144, Thr1145, Asp1266, and Leu1267 (Figure 1h). In a previous study, Hubbard et al. described the binding sites for IR phosphorylation at Tyr1158, Tyr1162, and Tyr1163, as well as the catalytic loop at Asp1132 and Asn1137.^87^ Thus, IEPGN presented interactions with Glu2 near the three-phosphorylation residues. The same trend was observed with PENQG (Pro1) and NIYQIS (Tyr3), both of which interacted with Tyr residues involved in INSR phosphorylation. Remarkably, in QDQHQKIR, three residues (His4, Gln5, and Lys6) interacted with residues within the phosphorylation cavity. Given that the quinoa peptides interacted with phosphorylation INSR residues and cavities, they may play a role in initiating the signaling pathway for GLUT4 receptor translocation.^83,84^

ACE-I is a carboxylase involved in the renin–angiotensin system (RAS), which affects cardiovascular function, blood pressure, renal filtration, hematopoiesis, inflammation, and immunity.^88–90^ Hence, the importance of inhibiting ACE-I (PDB: 1O8A) using quinoa peptides. In this regard, IEPGN showed the lowest free energy (−124.299 kcal/mol) and a smaller number of interactions, with four hydrogen bonds at Ala356, His383, Glu384, and His387. Moreover, PENQG showed five hydrogen bond interactions at Asn66, Asn70, His353, Lys368, and Tyr523, with a free energy of −144.254 kcal/mol. Then, QDQQF showed seven hydrogen bonds at Asn66, Asn70, Leu139, Ser355, Ala356, Tyr360, and His387. Notably, PENQG and QDQQF presented similar residue interactions (Asn66 and Asn70), both peptides are negatively charged and share similar characteristics (Table 1). Furthermore, YDDER (−159.079 kcal/mol Δ*G*) also exhibited seven hydrogen bonds at Tyr62, Lys118, Asp121, Glu123, Arg124, Ser516, and Ser517 (Figure 1i). The same behavior was observed for NIYQIS (Tyr51, Asn66, Thr92, Lys118, Asp121, Ser355, and Ala356) and QDQHKIR (Asn66, Asp358, Tyr360, Tyr394, Arg402, Gly404, and Arg522), both of which exhibited seven hydrogen bond interactions (Figure 1j).

Lisinopril and enalapril are known inhibitors of the ACE enzyme, with binding site residues located at Glu162, Arg186, Tyr224, His353, Ala354, Glu384, Arg489, His387, Glu411, Val518, Lys511, Arg522, and Tyr523.^81,91^ Notably, the quinoa peptides IEPGN (Glu384 and His387) and PENQG (His352 and Tyr523) presented two hydrogen bond interactions, which are also found in ACE-I drug inhibitors. Additionally, QDQQF (His387) formed one hydrogen bond similar to the standard inhibitor lisinopril. In general, the six quinoa peptides interacted with residues within the ACE-I cavity.

In general, lipoxygenases catalyze polyunsaturated fatty acids and are responsible for the inflammation pathway.^92,93^ Furthermore, lipoxygenases have a wide range of bioactive lipid mediators—leukotrienes, lipoxins, hepoxilins, eoxins, and protectins—and interact with pro-inflammatory factors.^93,94^ Hence, the relevance of inhibiting the lipoxygenase (PDB: 1N8Q) enzyme. Thus, IEPGN showed the lowest free energy, −110.892 kcal/mol, with four hydrogen bonds at Arg378, Arg386, Asp428, and Asp43. Furthermore, PENQG (−131.979 kcal/mol) presented five hydrogen bonds at Asn375, Arg378, Asn521, Val594, and Gln598. Additionally, both peptides exhibited the same interaction at Arg378 and presented an affinity for Glu (E) and Gly (G) in the peptide sequence. Moreover, QDQQF (−142.414 kcal/mol Δ*G*) exhibited four hydrogen bonds at Val128, Ser129, Thr131, and Asn788. Conversely, YDDER (−144.274 kcal/mol Δ*G*) showed the lowest hydrogen bond interactions, with only two at Tyr394 and Glu584. Interestingly, NIYQIS exhibited six hydrogen bonds at Thr91, Ala94, Gln96, Ser129, Pro789, and Asn790 (Figure 1k). The same trend was observed with QDQHKIR in both energy (−161.546 kcal/mol Δ*G*) and the number of hydrogen bond interactions (Pro435, Tyr436, Arg439, Arg580, Tyr581, and Glu584) (Figure 1l).

Quercetin is a well-described lipoxygenase inhibitor, with a central cavity in Gln514, and the molecule shifts to Leu277, Ile557, and Leu773.^95^ Even though quinoa peptides did not interact with the central cavity, some residues interacted close to them. Thus, PENQG (Gly5) interacted with the Ile557 active site. Moreover, QDQQF (Gln4) interacted with the Leu773 residue, while in the NIYQIS peptide, two amino acids (Gln4 and Ile5) interacted with this residue.

Overall, the MD results of this study suggest a potential therapeutic effect of quinoa-derived peptides (IEPGN, PENQG, QDQQF, YDDER, NIYQIS, and QDQHQKIR). Notably, QDQHQKIR demonstrated the strongest binding affinity to DPP-IV (−219.367 kcal/mol), forming multiple hydrogen bonds with key active site residues (e.g., Asn51, Glu205, and Asp545). These interactions underscore its potential role as a competitive inhibitor involved in glucose regulation. Based on these findings, the peptide–protein complexes with the highest binding affinities for each target were selected for MDS to further investigate their stability and interaction profiles.

### 3.4 Molecular Dynamics

Molecular dynamics simulations (MDS) were conducted to assess the stability of predicted interactions between peptides (IEPGN, PENQG, QDQQF, YDDER, NIYQIS, and QDQHQKIR) and target proteins, including α-glucosidase, DPP-IV, INSR, ACE-I, and lipoxygenase. Overall, the trajectory analysis was run for 40 ns. The RMSD trajectory for the complexes DPP-IV-NIYQIS and DPP-IV-QDQHQKIR reached equilibrium after 3 ns of MDS. Both peptides stabilize the protein and remained bonded in the interacting site (Figure 2,1E). The RMSF for both complexes showed a similar fluctuation pattern, which is in agreement with the RMSD trajectory (Figure 2,1F). Both peptides contain Gln (Q), a polar and neutral residue, as well as Ser (S) and His (H), which can form hydrogen bonds. Additionally, both have Ile (I), suggesting similar flexibility and the ability to participate in hydrogen bonding. The RMSD trajectory analysis of the ACE-I-QDQHQKIR complex reaches equilibrium after 10 ns of MDS, with ± 1 Å of increase throughout the entire trajectory (Figure 2,2E). In contrast, the ACE-I-YDDER complex reaches equilibrium after 5 ns and remains stable for the rest of the trajectory (Figure 2,2E). The RMSF trajectory reveals a similar fluctuation pattern for both systems; however, the ACE-I-QDQHQKIR complex exhibits higher fluctuation peaks than ACE-I-YDDER, which may influence the RMSD analysis (Figure 2,2F). Furthermore, the RMSD trajectory of the lipoxygenase-NIYQIS complex reaches the equilibrium after 10 ns of MDS and increases by 1 Å between 10 and 20 ns. After 20 ns, it remains unchanged for the rest of the simulation (Figure 2,3E). Meanwhile, the lipoxygenase-QDQHQKIR complex reaches equilibrium after 15 ns and remains stable for the rest of the trajectory (Figure 2,3E). Despite both peptides exhibiting similar behavior, lipoxygenase-NIYQIS system appears more stable than lipoxygenase-QDQHQKIR, where the trajectory increases by ±1 Å. The RMSF shows a similar fluctuation pattern for both systems (Figure 2,3F).

**Figure 2:**
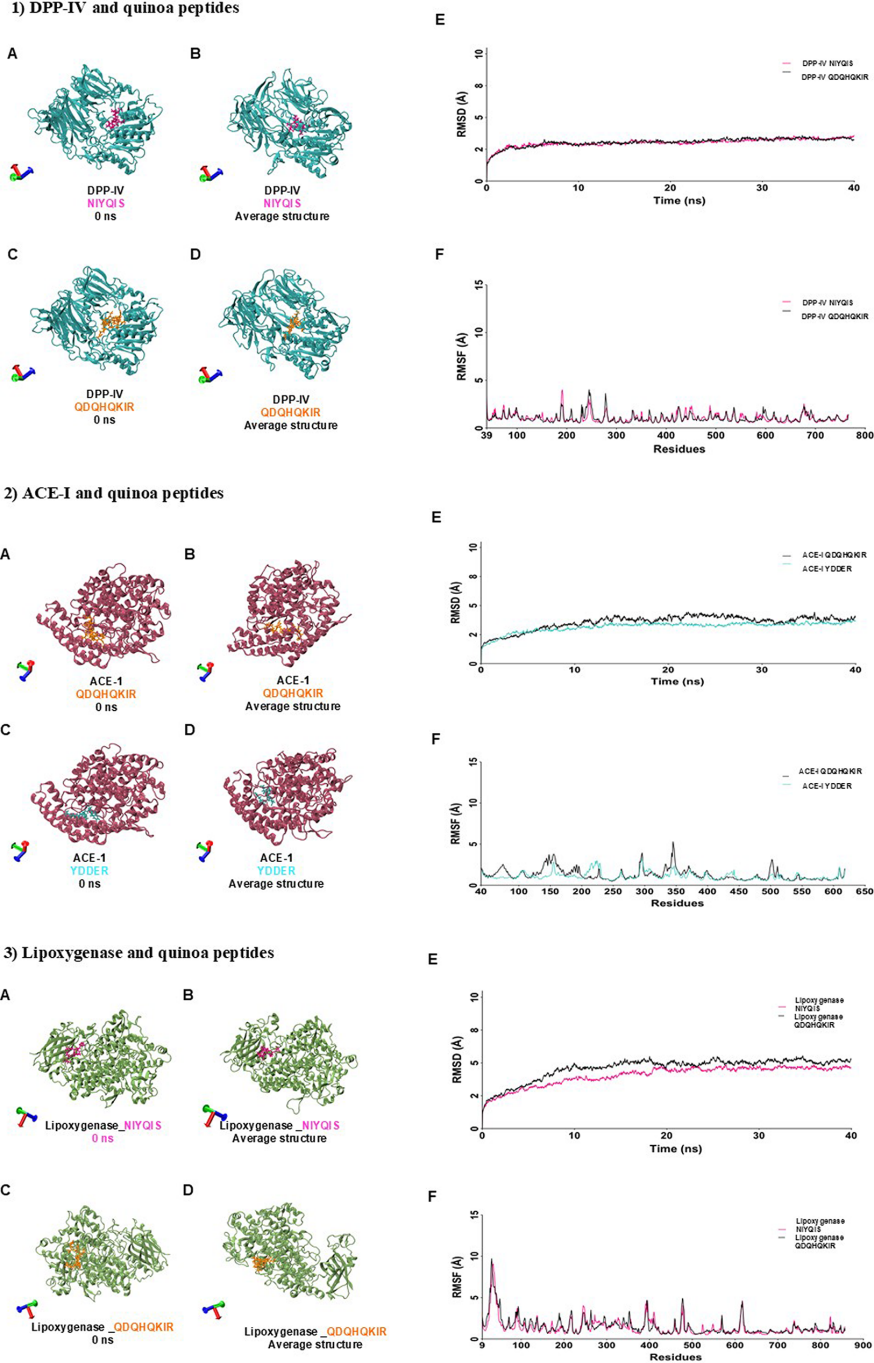
Molecular dynamics. 1A: DPP-IV with NIYQIS at 0 ns, 1B: DPP-IV with NIYQIS average structure,1C: DPP-IV with QDQHQKIR at 0 ns,1D: DPP-IV with QDQHQKIR average structure, 1E: RMSD of DPP-IV with NIYQIS and QDQHQKIR, 1F: RMSF of DPP-IV with NIYQIS and QDQHQKIR,2A: ACE-I with QDQHQKIR at 0 ns, B: ACE-I with QDQHQKIR average structure, 2C: ACE-I with YDDER at 0 ns, D: ACE-I with YDDER average structure, 2E: RMSD of ACE-I with QDQHQKIR and YDDER, 2F: RSMF of ACE-I with QDQHQKIR and YDDER, 3A: lipoxygenase with NIYQIS at 0 ns, 3B: lipoxygenase with NIYQIS average structure, and 3C: lipoxygenase with QDQHQKIR at 0 ns, 3D: lipoxygenase with QDQHQKIR average structure, 3E: RMSD of lipoxygenase with NIYQIS and QDQHQKIR, and 3F: RMSF of lipoxygenase with NIYQIS and QDQHQKIR.

Overall, quinoa peptides—YDDER, NIYQIS, and QDQHQKIR—were considered highly active and were chemically synthesized for antidiabetic, hypotensive and antioxidant *in vitro* activity. The MS/MS spectra of quinoa peptides are shown in Figure S3.

### 3.5 *In Vitro* Antidiabetic Activity

T2DM is a disease characterized by insulin resistance, hyperglycemia, and metabolic syndrome.^96^ As therapeutic peptides are gaining attention as an option to manage T2DM, this study aimed to analyze three quinoa peptides (YDDER, NIYQIS, and QDQHQKIR) in different enzymes (α-amylase, α-glucosidase, and DPP-IV) responsible for hyperglycemia control (Table 4).

**Table 4:** Oxygen Radical Absorbance Capacity (ORAC), and Inhibitory Activity of α-Amylase, α-Glucosidase, Dipeptidyl peptidase-IV (DPP-IV), and Angiotensin I Converting Enzyme (ACE-I) of Quinoa Peptides: YDDER, NIYQIS, and QDQHQKIR[Table-fn tbl4fn1]

	ORAC	α-amylase			α-glucosidase			DPP-IV			ACE-I		
Peptide	μM TE/μM Peptide	300 μM	400 μM	500 μM	300 μM	400 μM	500 μM	125 μM	250 μM	500 μM	125 μM	250 μM	500 μM
YDDER	0.24 ± 0.01^b^	12.20 ± 1.67^c^	11.43 ± 2.04^c^	6.66 ± 0.38^d^	8.48 ± 0.94^c^	8.33 ± 0.94^c^	14.86 ± 0.89^a^	ND	ND	3.44 ± 0.50^d^	21.1 ± 0.19^cd^	24.1 ± 0.18^c^	24.3 ± 0.15^c^
NIYQIS	0.75 ± 0.02^a^	ND	ND	ND	7.72 ± 0.95^c^	10.38 ± 0.93^bc^	11.93 ± 0.92^b^	ND	12.22 ± 0.50^b^	16.36 ± 0.70^a^	17.20 ± 0.28^d^	31.7 ± 2.40^b^	53.0 ± 1.41^a^
QDQHQKIR	0.02 ± 0.00^c^	18.43 ± 0.33^a^	15.61 ± 0.34^ab^	15.37 ± 0.35^b^	ND	ND	ND	ND	ND	5.26 ± 0.30^c^	6.8 ± 0.56^f^	9.75 ± 0.21^ef^	12.10 ± 0.99^e^

1 Different superscript letters (a-f) in the same assay indicate statistical differences between quinoa peptides—YDDER, NIYQIS, and QDQHQKIR—by one-way ANOVA and Tukey’s multiple range test. Data are expressed as mean ± SD, *n* = 9, (*p* < 0.05). ND: no inhibitory effect was observed at the given concentration.

#### 3.5.1 α-Amylase Inhibitory Activity

As α-amylase plays a significant role in T2DM, this study investigated the inhibitory effects of synthetic quinoa peptides on this enzyme. Among the tested peptides, QDQHQKIR presented the highest α-amylase inhibition with 18.43 ± 0.33% at 300 μM, followed by YDDER (12.20 ± 0.33% at 300 μM. Conversely, NIYQIS did not show significant α-amylase inhibition, even at the highest tested concentration (500 μM) (Table 4). In a previous study, Zhou et al. reported that quinoa-derived peptides with short length (less than six amino acids) and high hydrophobicity showed stronger α-amylase inhibition, as demonstrated by peptide MMFPH.^70^ Different studies have reported that peptide sequences containing Trp, Arg, and Tyr at the C-terminal have a higher probability of binding to the active site cavity of α-amylase, enhancing their inhibitory effects.^30,70^ In this regard, QDQHQKIR has an Arg residue at the C-terminus, impairing the catalytic function of α-amylase and contributing to its significant inhibitory effect.

#### 3.5.2 α-Glucosidase Inhibitory Activity

The inhibitory effects of quinoa-derived peptides (YDDER, NIYQIS, and QDQHQKIR) toward α-glucosidase are depicted in Table 4. Among the tested peptides, YDDER exhibited the highest inhibition (14.86 ± 0.89% at 500 μM), followed by NIYQIS (11.93 ± 0.89% at 500 μM), with a significant difference between them (*p* < 0.05). In contrast, QDQHQKIR did not present any α-glucosidase inhibition at any of the tested concentrations (100–500 μM). Comparatively, Vilcacundo et al. reported a significantly higher α-glucosidase inhibition of 55.85% (250 μM) for IQEGGLT quinoa-derived peptide, mainly containing hydrophobic and neutral residues.^40^ In this context, NIYQIS has a distribution of neutral and hydrophobic residues. Additionally, the Arg residue in the C-terminal and the number of Asp residues in YDDER might be key factors contributing to its α-glucosidase inhibition. In general, aromatic residues (e.g., Tyr and Phe), along with basic residues (e.g., Lys, Arg, and His), have been shown to play a pivotal role in binding to α-glucosidase active site, either by forming hydrogen bonds or hydrophobic interactions.^30,97^ Notably, YDDER showed inhibitory activity against both α-amylase and α-glucosidase, further highlighting its biological potential as a multifunctional peptide for glucose regulation.

#### 3.5.3 DPP-IV Inhibitory Activity

The inhibition of DPP-IV not only increases GLP-1 during the prandial stage but also has sustained metabolic effects over 24 h period.^98^ In this study, NIYQIS exhibited the highest DPP-IV inhibition (16.36 ± 0.70% at 500 μM), while QDQHQKIR (5.26 ± 0.70% at 500 μM) and YDDER (3.44 ± 0.70% at 500 μM) showed slight inhibition. No inhibitory activity was detected at 125 μM. A previous study by Vilcacundo et al. reported that the DPP-IV quinoa-derived peptide IQAQGGLT exhibited 17.05% inhibition at 250 μM.^40^ The literature review suggests that specific amino acid residues in the N-terminal position (Leu, Ile, Val, His, Phe, Trp, and Tyr) are essential for DPP-IV inhibition.^40,76,99^ Nongonierma and Fitzgerald described that hydrophobic amino acids exhibited higher DPP-IV inhibition, compared to hydrophilic ones, suggesting that hydrophobic residues might enhance the peptide activity.^79,100^ In this study, NIYQIS did not show any of the expected residues in the N-terminal associated with strong inhibition, yet it has significant hydrophobic content. Overall, additional features such as residue positioning, peptide length, or structural conformation may play a role in determining peptide activity.

### 3.6 *In Vitro* Antioxidant Activity

Antioxidants inhibit oxidative pathways through various mechanisms, including peroxide inactivation, scavenging of free radicals, metal chelation, inactivation of reactive oxygen species (ROS), and lipid oxidation.^101–103^ Previous studies have found that food-derived peptides function as metal chelators, reducing agents, free radical scavengers, and ROS scavengers.^101,102,104–106^ For this reason, quinoa peptides YDDER, NIYQIS, and QDQHQKIR were analyzed using multiple antioxidant assays; the results are shown in Figure 3 and Table 4.

**Figure 3:**
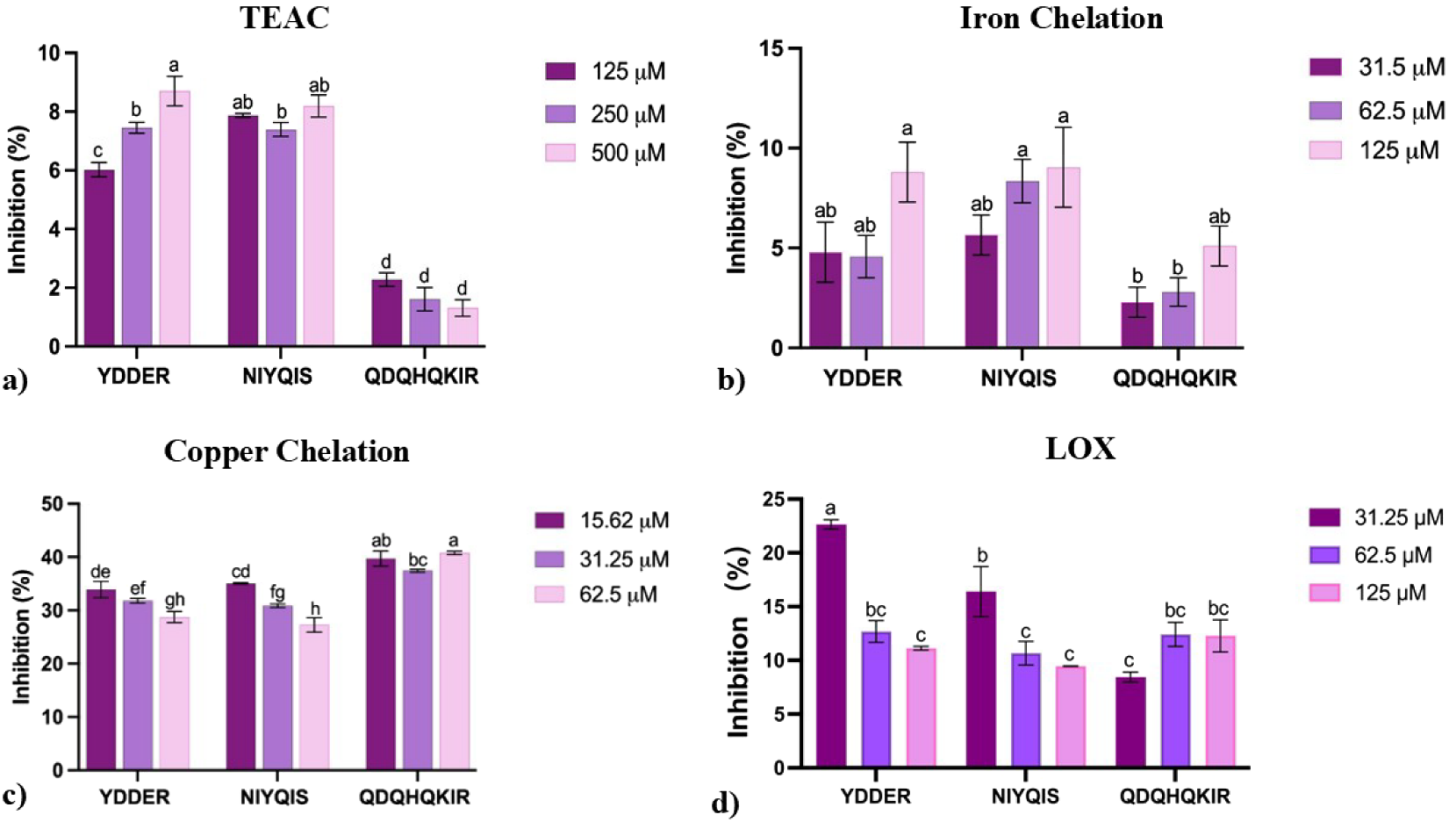
Antioxidant activity of quinoa peptides. (a) Trolox equivalent antioxidant capacity (TEAC), (b) iron chelation, and (c) copper chelation and lipoxygenase inhibition (LOX). Different superscript letters between bars indicate statistical analysis differences between quinoa peptides by one-way ANOVA and Tukey’s multiple range test. Data are expressed as mean ± SD, *n* = 9 (*p* < 0.05).

#### 3.6.1 ORAC

The ORAC method is particularly relevant as it mimics oxidative stress under physiological conditions.^101^ The results of the inhibition of ORAC by YDDER, NIYQIS, and QDQHQKIR are depicted in Table 4. In this context, NIYQIS exhibited the highest ORAC inhibition (0.75 μmol TE/μmol peptide, *p* < 0.05). YDDER exhibited a mild inhibition (0.24 μmol TE/μmol peptide, *p* < 0.05), while QDQHQKIR showed the lowest ORAC inhibition (0.02 μmol TE/μmol peptide, *p* < 0.05). Extensive literature has shown that hydrophobic residues and specific amino acid residues in the N-terminal (Cys, Met, Tyr, and His) exhibit higher antioxidant capacity.^12,107^ In this context, YDDER lacks hydrophobic residues but contains Tyr at the N-terminal, which may contribute to its antioxidant potential. In contrast, NIYQIS is 33.33% hydrophobic due to the presence of two Ile residues. Amino acids such as Cys, Phe, Leu, and Ile, as well as hydrophobic residues, are reported to increase the antioxidant capacity of peptides.^12,101,104^

Overall, the antioxidant capacity of a peptide relies on its amino acid sequence, spatial structure, MW, and chemical features.^102,108^

#### 3.6.2 TEAC

The TEAC inhibition (%) of quinoa-derived peptides is depicted in Figure 3a. Among all tested peptides, YDDER displayed the highest ABTS^●+^ inhibition, with values of 8.7 ± 0.50% at 500 μM and 7.45 ± 0.19% at 250 μM, showing a dose-dependent trend. Similarly, NIYQIS demonstrated ABTS^●+^ inhibition values of 8.19 ± 0.38% at 500 μM, 7.39 ± 0.19% at 250 μM, and 7.87 ± 0.10% at 125 μM. Notably, no statistical difference (*p* > 0.05) was observed between YDDER at 500 μM, NIYQIS at 500 μM, and NIYQIS at 250 μM. Conversely, QDQHQKIR displayed the lowest TEAC inhibition values, ranging from 1.31 to 2.28% across all tested concentrations. In a previous study by Wang et al. reported that Trp or Tyr residues at the C-terminal are strongly associated with radical-scavenging activity, as Trp can function as a hydrogen donor in oxidation reactions.^109^ Similarly, Yu et al. reported that aromatic residues (e.g., Tyr and Phe) could be considered as radical scavenger peptides.^110^ Furthermore, multiple studies revealed that hydrophobic residues (e.g., His, Val, Leu, Ile, or Ala) at the C-terminal contribute to strong antioxidant capacity in TEAC inhibition.^103,108,109,111^ In this study, only NIYQIS presented a significant hydrophobic composition, which may explain its higher TEAC inhibition compared to QDQHQKIR. These findings highlight the importance of overall residue composition and sequence arrangement in determining antioxidant capacity, beyond just the presence of specific residues in the N-terminal and C-terminal.^101,103,112^

#### 3.6.3 Metal Chelation (Fe^2+^ And Cu^2+^)

In the ferrous iron chelating assay (Fe^2+^ chelation), the reduction of Fe^3+^ to Fe^2+^ is determined using the ferrozine compound, a highly sensitive method that can be correlated with the structure–activity of the antioxidant compounds.^112,113^ The metal chelating activity of quinoa peptides is depicted in Figure 3b,*c. Quinoa* peptides showed a relatively low Fe^2+^ chelating activity (Figure 3b). Among them, NIYQIS showed the highest inhibition (9.04 ± 0.96% at 125 μM), followed by YDDER (8.8 ± 1.0% at 125 μM) and QDQHQKIR (5.11 ± 1.0% at 125 μM). No statistically significant differences (*p* > 0.05) were observed among the peptides at a concentration of 125 μM. Additionally, all peptides presented a dose-dependent response. A previous study by Wang et al. reported that peptides with a MW of 5–10 kDa exhibited stronger chelating capacity than peptides with MW < 3 kDa.^109^ In general, Fe^2+^ chelation is lower compared to Cu^2+^ chelation activity, as observed in previous studies.^42^

Copper plays an essential role in cell metabolism, oxidative stress regulation, and the biosynthesis of neurotransmitters.^113,114^ However, excess Cu^2+^ can participate in ROS formation via the Fenton-like reaction, leading to oxidative damage in biological tissues.^115^ The Cu^2+^ chelating activity of YDDER, NIYQIS, and QDQHQKIR is presented in Figure 3c. Interestingly, QDQHQKIR showed the highest activity, with values of 40.8 ± 0.31% (62.5 μM), 37.46 ± 0.26% (31.25 μM), and 39.79 ± 1.4% (15.62 μM). It is important to highlight that only QDQHQKIR exhibited a dose-dependent response, while YDDER and NIYQIS showed a decrease in chelating activity at higher concentrations. Furthermore, these peptides showed similar Cu^2+^ chelation activity (%), with no statistical difference (*p* > 0.05) observed at the lowest concentration (15.62 μM), potentially limiting their maximum chelation activity.

Different studies have identified specific amino acids associated with metal chelation, particularly Asp, Glu, Tyr, Trp, basic residues (e.g., Lys, His, and Arg), and hydrophobic residues (e.g., Leu, Ile, Val, and Pro) in the C-terminal, which likely contribute to metal chelation activity.^101,116^ A previous study reported the importance of the third amino acid residue in a peptide sequence in determining the chelating activity. Particularly, peptides containing Trp at the third position exhibited increased chelating activity, while Arg, Pro, Gly, and Thr at the same position decreased chelating activity.^117^ In this context, NIYQIS exhibits Trp at the third residue and a significant hydrophobic character, which may contribute to its antioxidant and chelating properties. Among all quinoa peptides, NIYQIS consistently demonstrated strong antioxidant activity across multiple assays. This suggests the potential of this synthetic peptide as a therapeutic agent for managing oxidative stress and chronic diseases.

#### 3.6.4 LOX Inhibitory Activity

LOX are enzymes involved in the biosynthesis of inflammatory mediators such as leukotrienes and have been implicated in several diseases such as atherosclerosis, asthma, Alzheimer’s disease, obesity, T2DM, and cancer.^43,94^ Due to their role in these pathologies, the inhibition of LOX activity is considered a relevant therapeutic target.^43,118^ The LOX inhibition by quinoa-derived peptides—YDDER, NIYQIS, and QDQHQKIR—is displayed in Figure 3d. 15-LO from soybean was used as a positive control with an inhibition of 98 ± 1.0% (1 mM). YDDER exhibited the highest LOX inhibition at 22.64% (31.25 μM), followed by NIYQIS at 16.20% (31.25 μM). Conversely, QDQHQKIR showed the lowest LOX inhibition at 8.44% (31.25 μM). Notably, YDDER and NIYQIS showed an inverse trend, exhibiting higher inhibition rates with lower concentrations, this behavior was observed across antioxidant assays.

Ding et al. evaluated eight peptides from velvet antler blood (LFP, FPH, EHF, VGYP, FSAL, LSQKFPK, HHGGEFTPV, and LKECCDKPV) for anti-LOX activity.^119^ These peptides exhibited an overall ≤12% (1 mg/mL) of inhibition.^119^ Furthermore, LFP and FSAL exhibited the highest inhibitions of 10% and 12%, respectively. Comparable to these results, quinoa-derived peptides at 62.5 and 125 μM also exhibited low inhibition, reinforcing the observation that LOX inhibition by food-derived peptides tends to be modest. Karas et al. reported six LOX-inhibitory peptides from millet grain (RLARAGLAQ, YGNPVGGVGH, EQGFLPGPEESGR, GQLGEHGGAGMG, GNPVGGVGHGTTGT, and GEHGGAGMGGGQFQPV).^120^ A notable common feature of these peptides was the presence of at least one glycine (G) residue; thus, the highest LOX inhibition was exhibited by GQLGEHGGAGMG (50%). The authors suggest that glycine-rich peptides might exhibit a potent anti-LOX effect.^120^ Conversely, the quinoa-derived peptides in this study do not have this feature, thus attributing a mild to low anti-LOX effect. A possible hypothesis is that the presence of Tyr or Asn at the N-terminus could be responsible for the mild anti-LOX activity. In this assay, there was no correlation with the MD results, as NIYQIS exhibited a high number of interactions with the protein (lipoxygenase), while YDDER only presented two interactions, and *in vitro* the opposite trend was found. To date, there is scarce information about LOX interaction with synthetic peptides, especially those derived from pseudocereal sources such as quinoa.^118^ Our findings contribute to this underexplored area and suggest a modest but measurable LOX-inhibitory effect from specific quinoa-derived peptides.

### 3.7 ACE-I Inhibitory Activity

The ACE-I inhibition (%) of quinoa-derived peptides (YDDER, NIYQIS, and QDQHQKIR) is presented in Table 4. As a reference, a commercial ACE-I inhibitor provided by the assay kit demonstrated an inhibition of 80.1% at 50 mM. Among the peptides, NIYQIS exhibited the highest inhibitory activity, with 53.0% at 500 μM and 31.7% at 250 μM. YDDER followed with a moderate inhibition of 24.3% at 500 μM, showing statistical differences between concentrations (*p* < 0.05). Conversely, QDQHQKIR showed the lowest ACE-I inhibitory activity, with concentrations of ≥12.10% at 500 μM. Moreover, NIYQIS and YDDER both feature polar amino acids (e.g., Tyr and Asn) at the N-terminal and overall neutral charges, which could explain their ACE-I inhibitory activity. Conversely, NIYQIS exhibited a significant hydrophobic component (33.3%, Table 1) in its sequence and possesses several Ile amino acids (aromatic). Although the MD showed that ACE-I inhibition could be higher in QDQHQKIR and YDDER, the *in vitro* analysis revealed that NIYQIS showed higher activity. This discrepancy again highlights the limitations of predictive modeling and reinforces the necessity of experimental validation.

Yudho-Sutopo et al. reported the ACE-I inhibitory activity of four synthetic peptides from pearl garlic (DHSTAVW, KLAKVF, KLSTAASF, and KETPEAHVF).^121^ The four peptides exhibited potent ACE-I inhibition with IC_50_ values of 2.8 (DHSTAVW), 77.2 (KLAKVF), 172.2 (KLSTAASF), and 455.4 μM (KETPEAHVF). According to Yudho-Sutopo et al., the hydrophobic amino acid in the C-terminal and amino acids with a positively charged side-chain amino group, like in DHSTAVW, are determinants in the inhibition of ACE-I.^121^ Furthermore, a study by Zheng et al. reported an ACE-I inhibitory peptide – RGQVIYVL – from *Chenopodium quinoa* Willd, with an IC_50_ value of 38.16 μM. This peptide has a strong hydrophobic residue content of 62.50%.^24^ The authors concluded that the presence of Tyr and branched-chain amino acids (e.g., Leu and Val) at the C-terminal, and Arg at the N-terminal could be responsible for the activity against ACE-I. In general, several studies have demonstrated that hydrophobic amino acid residues or aromatic residues in the C-terminal have a high affinity in the active site of ACE-I.^24,33,122^ Although the MD showed that ACE-I inhibition could be higher in QDQHQKIR and YDDER, the *in vitro* analysis revealed that NIYQIS showed a higher activity. Overall, our findings align with those of these prior studies and support the conclusion that specific structural features, especially hydrophobic and aromatic residues, play a significant role in ACE-I inhibition.

Overall, this study conducted a comprehensive bioinformatics and experimental analyses to explore the bioactive potential of quinoa-derived peptides obtained from the *in silico* hydrolysis of 11S-G (*Chenopodium quinoa* Willd) using stem bromelain (EC 3.4.22.32). A total of 109 peptides were generated, of which 14 sequences contained more than five amino acids. These peptides were predicted as nontoxic, mostly neutral, and exhibited strong protein – ligand interactions with key metabolic enzymes or receptors, including ACE-I, DPP-IV, α-glucosidase, INSR, and lipoxygenase, as demonstrated by molecular docking (MD) and molecular dynamics simulations (MDS).

Experimentally, QDQHQKIR showed the highest α-amylase inhibition and Cu^2+^ chelation rate, suggesting a promising role in carbohydrate metabolism regulation and metal ion homeostasis. Furthermore, YDDER exhibited the highest LOX inhibition, suggesting a possible anti-inflammatory effect. Remarkably, NIYQIS exhibited the highest inhibitory activity against α-glucosidase, DPP-IV, and ACE-I, as well as the strongest ORAC, highlighting its dual antioxidant and antidiabetic potential. These results aligned with *in silico* predictions, where NIYQIS had the highest binding affinity and hydrogen bond interactions with α-glucosidase and DPP-IV, further supporting its functional bioactivity.

Overall, NIYQIS emerged as the most promising peptide, displaying strong antioxidant and hypotensive properties, and moderate antidiabetic activity, while QDQHQKIR demonstrated superior enzymatic inhibition and metal chelation capacity. These findings suggest that quinoa-derived peptides have significant potential as functional ingredients for managing oxidative stress and metabolic disorders. Despite this, further research is needed to validate these bioactivities *in vivo*, investigate peptide stability, bioavailability, and absorption, and explore potential formulation strategies to enhance their delivery and efficacy in functional foods or therapeutic applications.

## Supplementary Material



## Notes

Figure S1: Different peptides released after the simulated hydrolysis of 11S seed globulin – *Chenopodium quinoa* (AAS67037.1) – with stem bromelain. Figure S2. Molecular docking, a general view of protein-ligand interactions showing the residues from the active site involved in making the interactions with the ligand (quinoa peptides).Figure S3: Mass spectrum analysis of quinoa-derived peptides (from simulated hydrolysis with stem bromelain) YDDER, NIYQIS, and QDQHQKIR after being chemically synthesized (PDF)

## Abbreviations

11S-G: seed globulin storage protein
ACE-I: angiotensin I converting enzyme
ADMET: absorption, distribution, metabolism, excretion, and toxicity
BBB: blood–brain barrier penetration
CYP: cytochrome 450
DM: diabetes mellitus
DPP-IV: dipeptidyl peptidase IV
FASTA: fast adaptive shrinkage threshold algorithm
GIP: gastric inhibitory polypeptide
GLP-1: glucagon-like peptide-1
GOPOD: glucose oxidase and peroxidase
HIA: human intestinal absorption
IR: insulin resistance
INSR: insulin receptor
LOX: lipoxygenase inhibitory activity assay
MD: molecular docking
MDS: molecular dynamics simulations
MW: molecular weight
NCDs: noncommunicable diseases
ORAC: oxygen radical absorption capacity
PBC: periodic boundary conditions
PBS: phosphate-buffered saline
PDB: protein data bank
PPA: porcine pancreatic α-amylase
PPB: plasma protein binding
RD: radius of gyration
RMSD: root-mean-square deviation
RMSF: root-mean-square fluctuation
RT: room temperature
SMILES: simplified molecular input line entry system
SVM: support vector machine
TEAC: trolox equivalent antioxidant capacity
T2DM: type 2 diabetes mellitus
