# Supplementary material for: Antioxidant, Hypotensive, and Antidiabetic Breakthroughs: Bromelain Hydrolysis Unlocks Quinoa’s Peptide Potential - In Silico and In Vitro Approach
Source: J Agric Food Chem. 2025 Aug 25;73(36):22877–94. doi: 10.1021/acs.jafc.5c03789 (PMC12426930; doi:10.1021/acs.jafc.5c03789)
Supplement: Supplementary file 1 [file jf5c03789_si_001.pdf]

Antioxidant, Hypotensive and Antidiabetic Breakthroughs: Bromelain Hydrolysis Unlocks Quinoa's Peptide Potential - *In Silico* and *In Vitro* Approach

Maria Lilibeth Manzanilla-Valdez[1,4], Sarita Montaño[2], Cristina Martinez-Villaluenga[3], María Fernanda Zúñiga-Ayala[2], Christine Boesch[1,4], and Alan Javier Hernandez-Alvarez[1,4*].

[1] School of Food Science and Nutrition, University of Leeds, LS2 9JT, Leeds, UK

[2] Laboratorio de Bioinformática y Simulación Molecular, Facultad de Ciencias Químico Biológicas, Universidad Autónoma de Sinaloa, 80030, Sinaloa, México.

[3] Department of Technological Processes and Biotechnology, Institute of Food Science, Technology and Nutrition (ICTAN-CSIC), Jose Antonio Novais 6, 28040, Madrid, Spain

[4]National Alternative Protein Innovation Centre (NAPIC), UK

*Corresponding author: a.j.hernandezalvarez@leeds.ac.uk

Table of Contents (TOC)

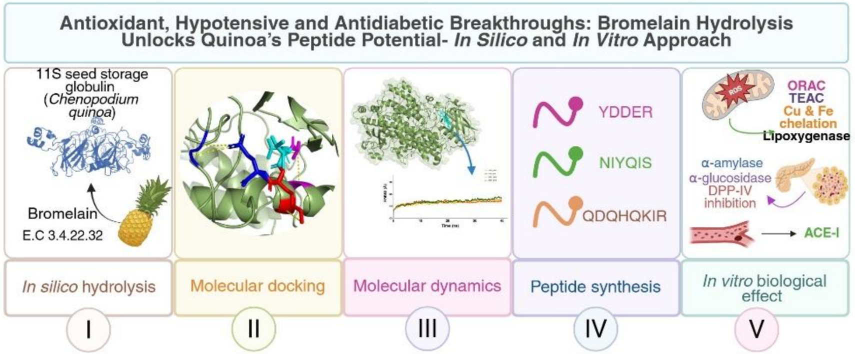

Supporting Information

Figure S1. Peptide released from 11S seed globulin *Chenopodium quinoa* (AAS67037.1) after simulated hydrolysis with stem bromelain.

Stem Bromelain (EC 3.4.22.32) hydrolysis

Results of enzyme action

QG - R - MR - EMQG - NECQIDR - L - T - A - L - EPT - NR - IQA - EG - G - L - T - EV - WDT - QDQQF - QCS - G - V - S - V - IR - R - T - IEPNG - L - L - L - PS - F -
T - S - G - PEL - IYIEQG - NG - IS - G - L - MIPG - CPET - F - ES - MS - QES - WR - EG - MKR - G - MR - G - G - R - F - QDQHQKIR - HL - R - QG - HIF - A - MPA
- G - V - A - HWA - YNT - G - NEPL - V - A - V - IL - IDT - S - NHA - NQL - DKDYPKR - F - YL - A - G - KPQQEHS - R - HQHR - G - G - ES - QR - G - ER - G - S - G
- G - NV - F - S - G - L - G - T - KT - IA - QS - F - G - V - S - EDIA - EKL - QA - EQDER - G - NIV - L - V - QEG - L - HV - IKPPS - S - R - S - YDDER - EQR - R - HR
- S - PR - S - NG - L - EET - ICS - A - R - L - S - ENIDEPS - KA - DV - YS - PEA - G - R - L - T - T - L - NS - F - NL - PIL - S - NL - R - L - S - A - EKG - V - L - YR -
NA - IMA - PHYNL - NA - HS - IIYG - V - R - G - R - G - R - IQIV - NA - QG - NS - V - F - DDEL - R - QG - QL - V - V - V - PQNF - A - V - V - KQA - G - EEG - F -
EWIA - F - KT - CENA - L - F - QT - L - A - G - R - T - S - A - IR - A - MPL - EV - IS - NIYQIS - R - EQA - YR - L - KF - S - R - S - ET - T - L - F - R - PENQG - R - QR
- R - DL - A - A

Figure S2. Molecular docking, a general view of protein-ligand interactions showing the residues from the active site involved in making the interactions with the ligand (quinoa peptides).

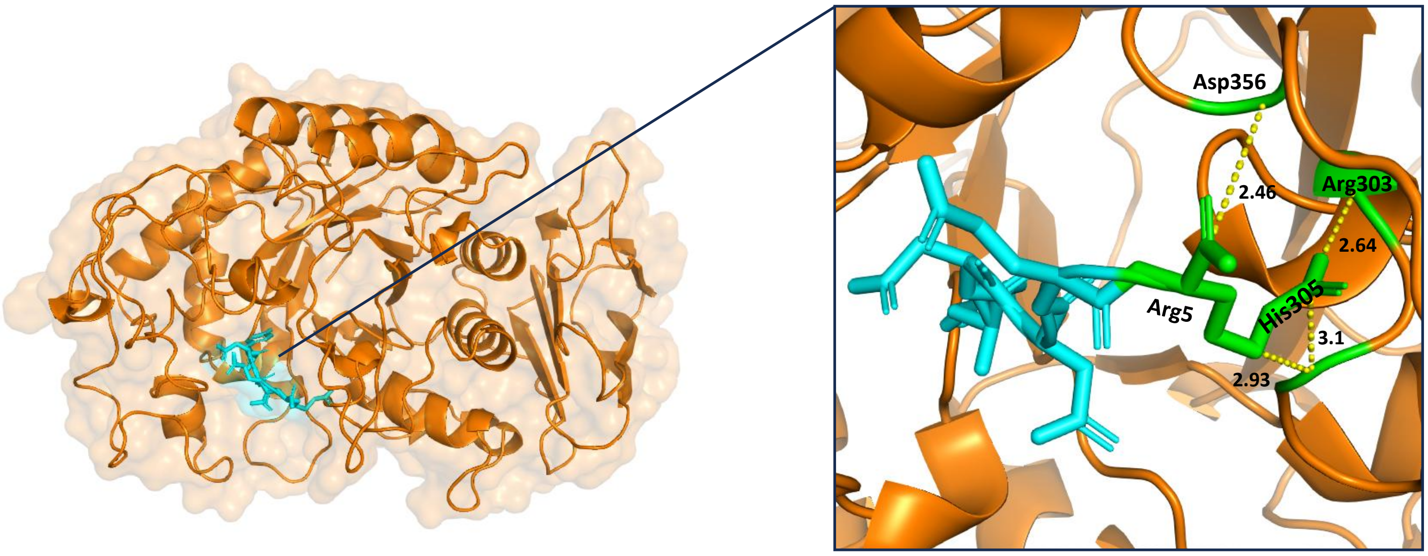

a) YDDER with α-amylase

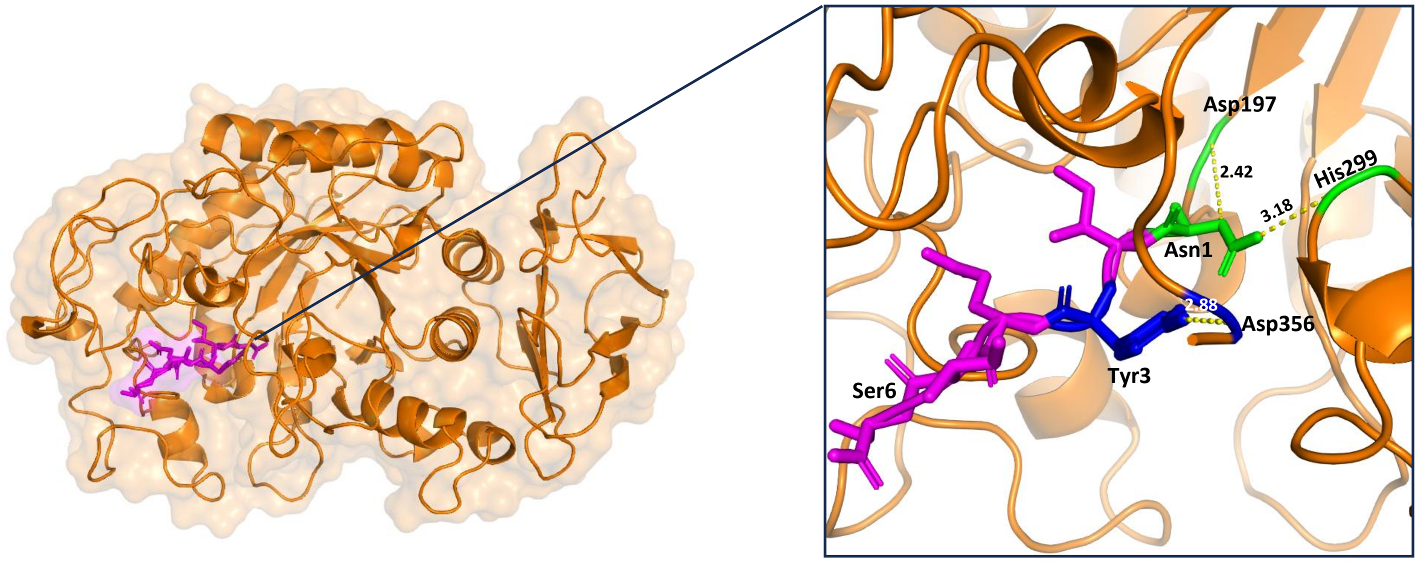

b) NIYQIS with α-amylase

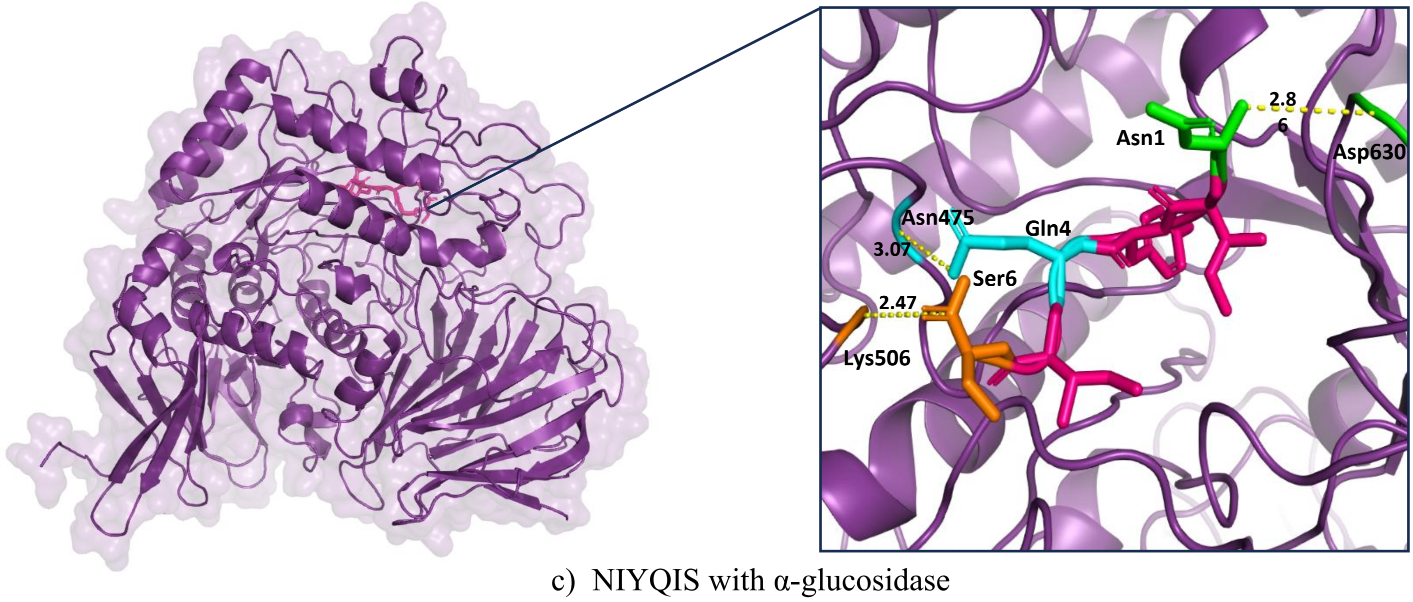

c) NIYQIS with α-glucosidase

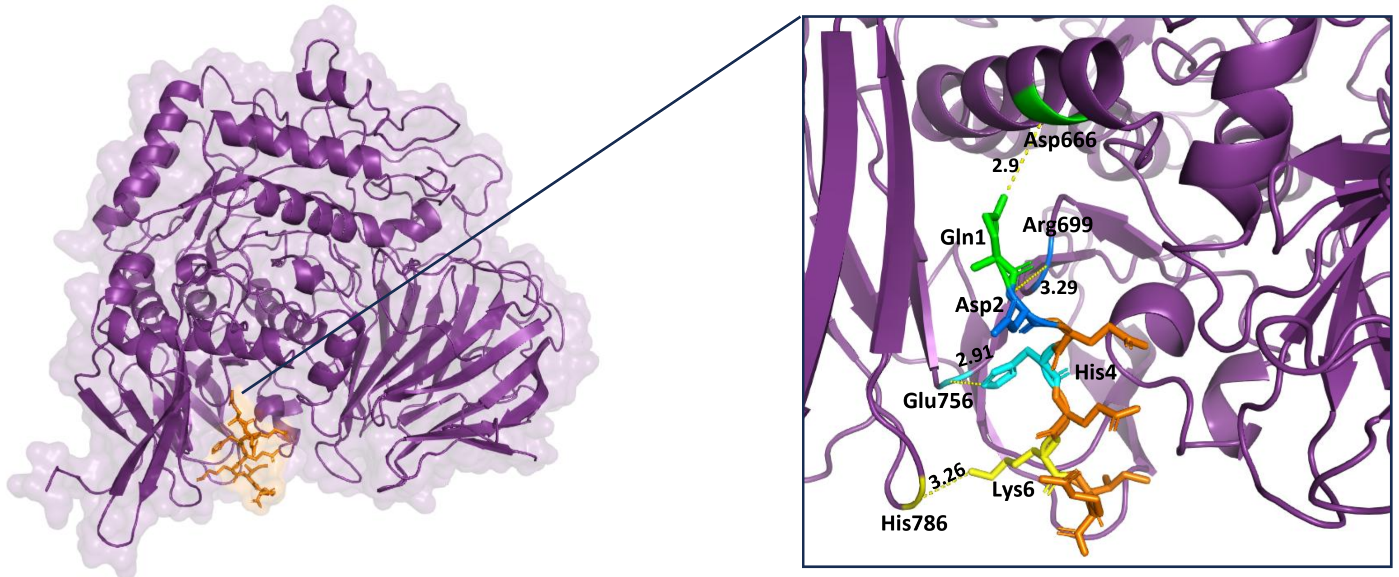

d) QDQHKIR with α-glucosidase

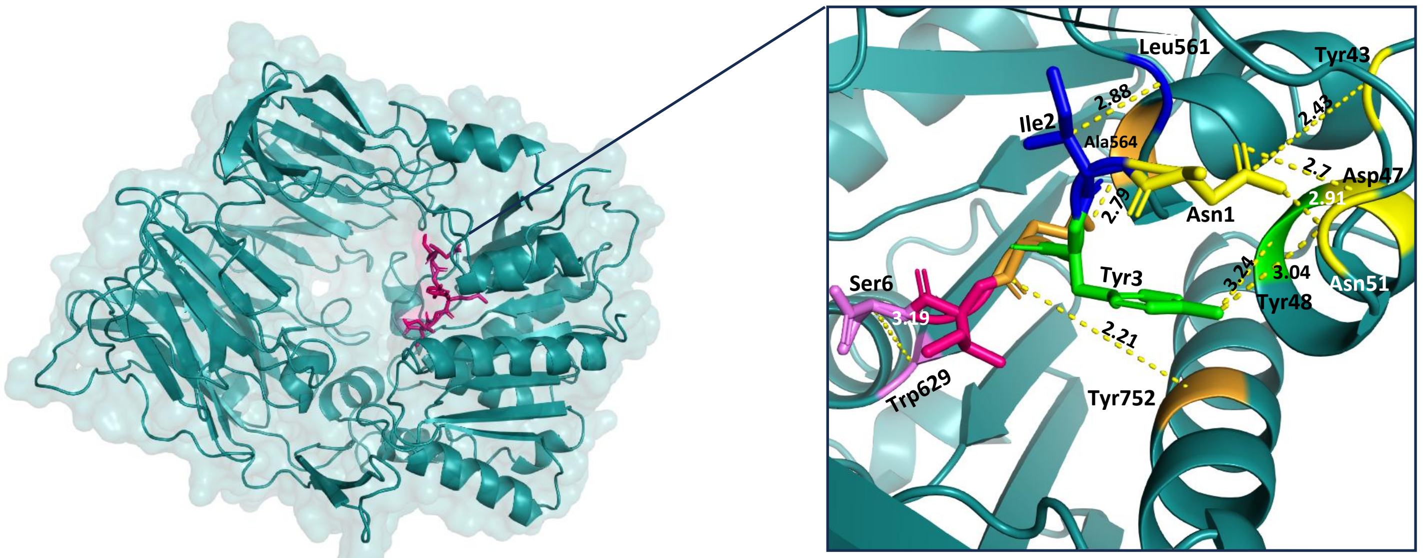

e) NIYQIS with DPP-IV

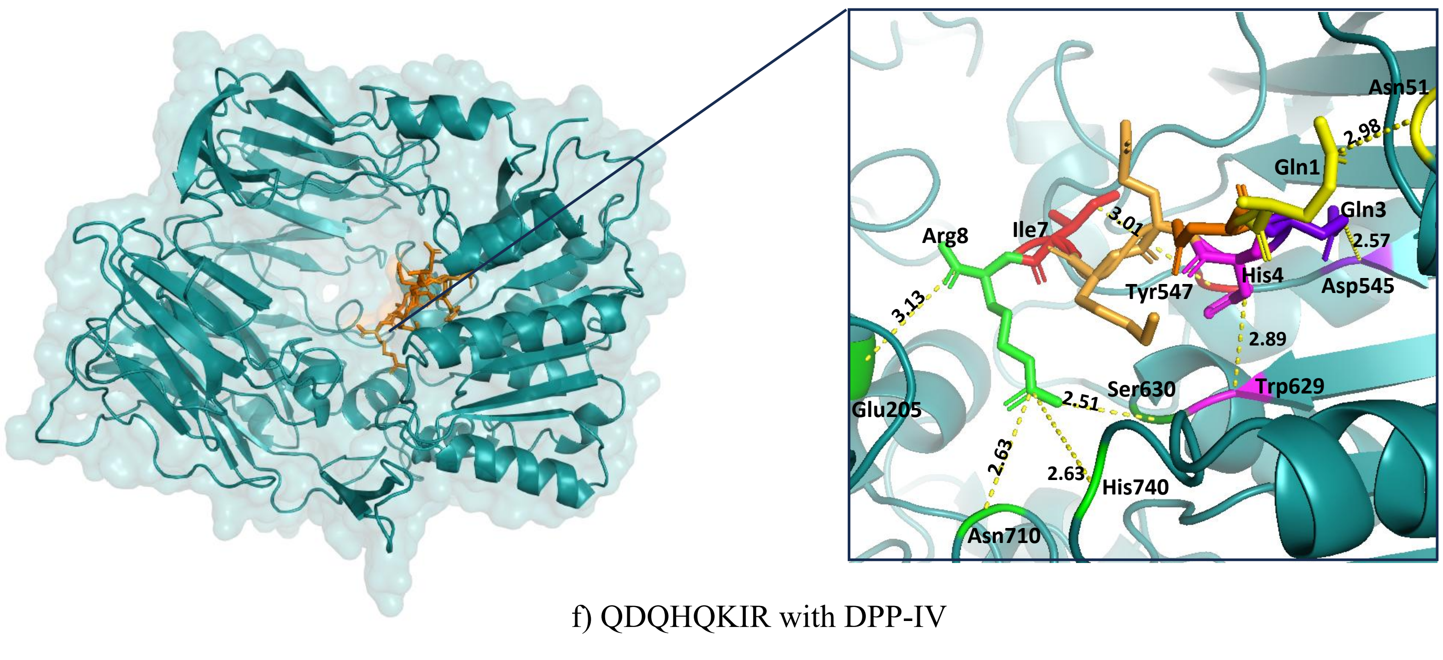

f) QDQHQKIR with DPP-IV

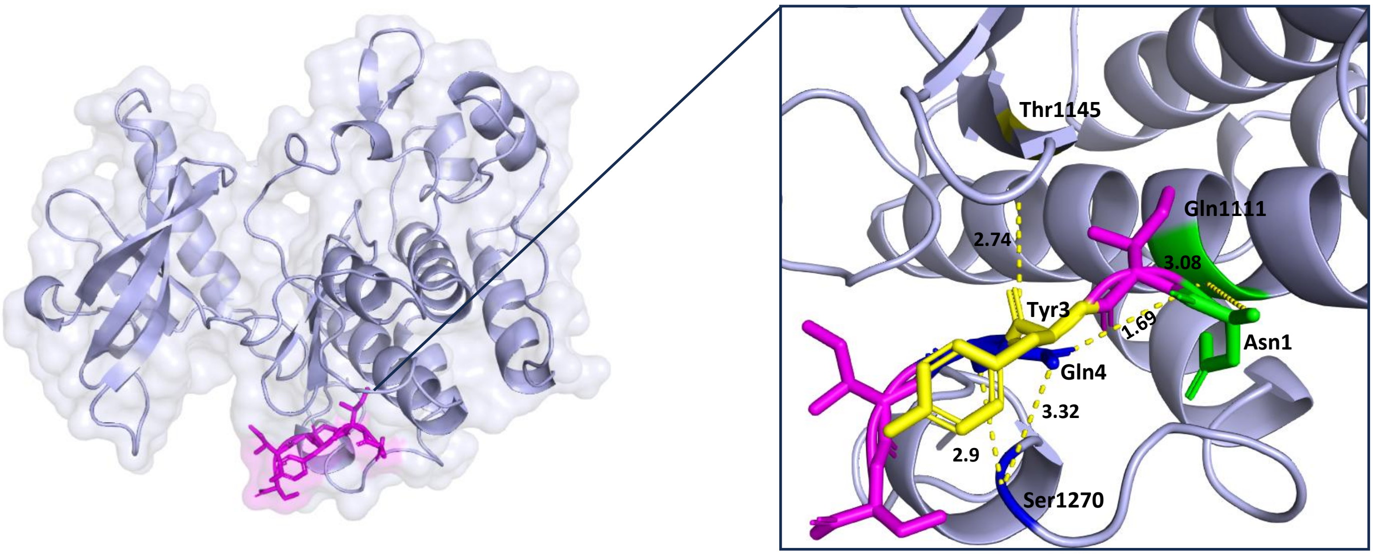

g) NIYQIS with INSR

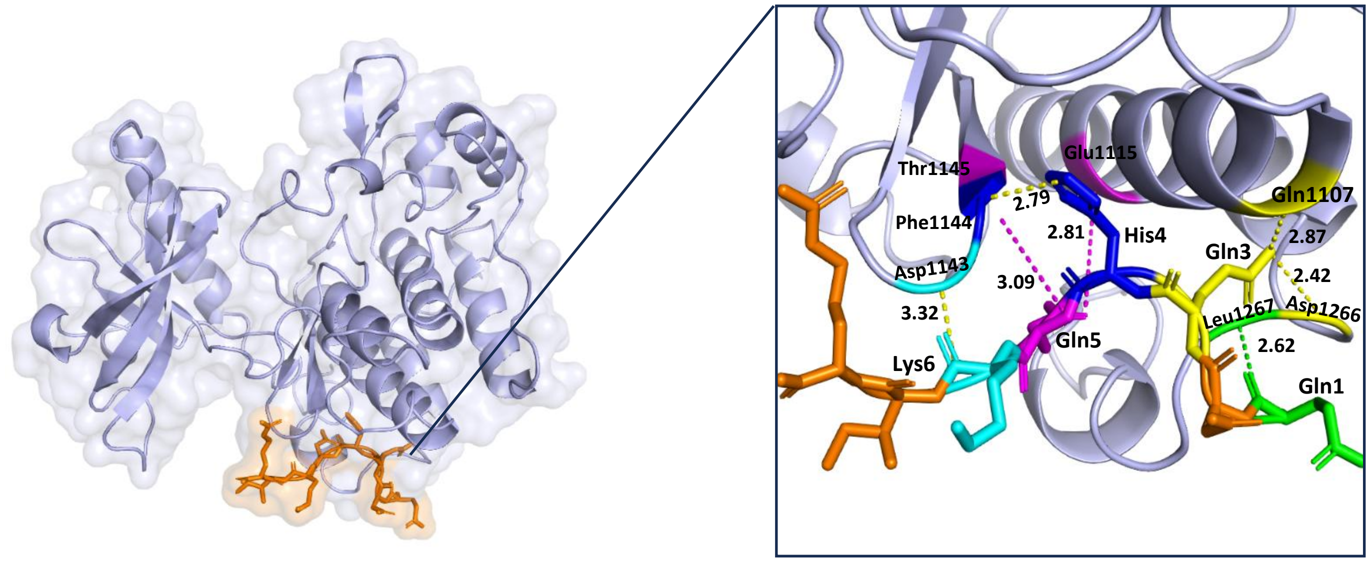

h) QDQHQKIR with INSR

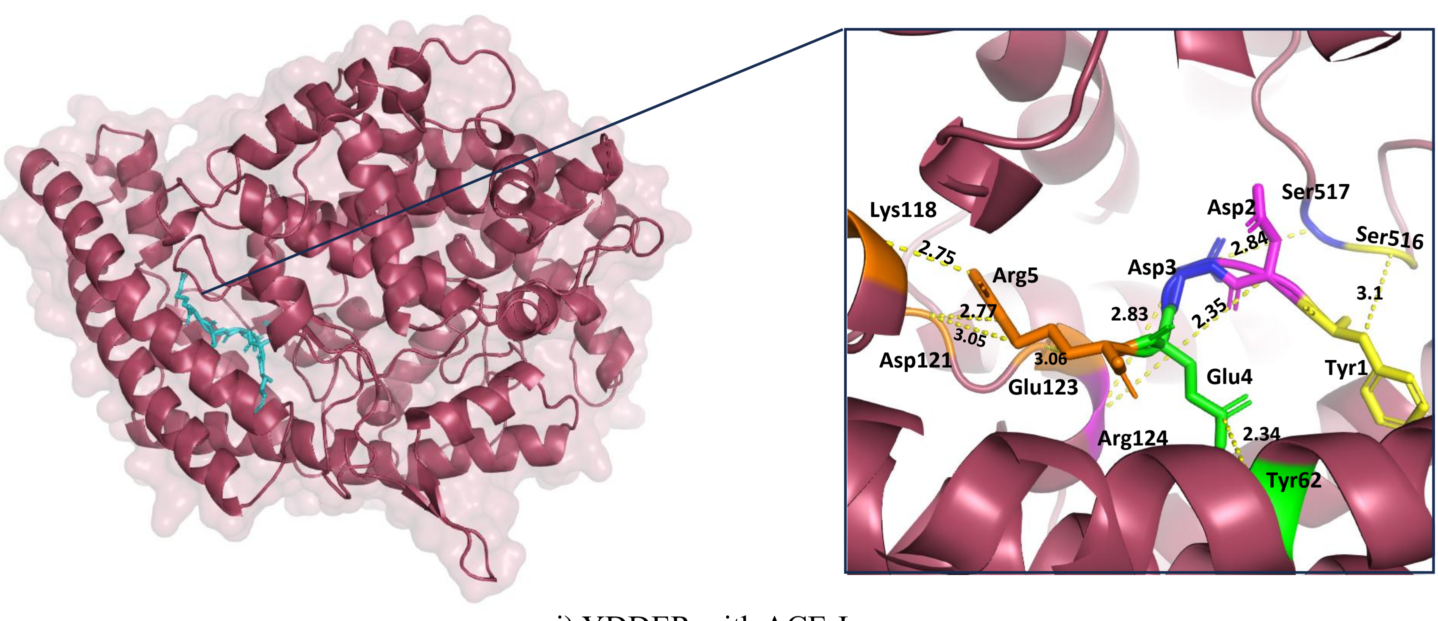

i) YDDER with ACE-I

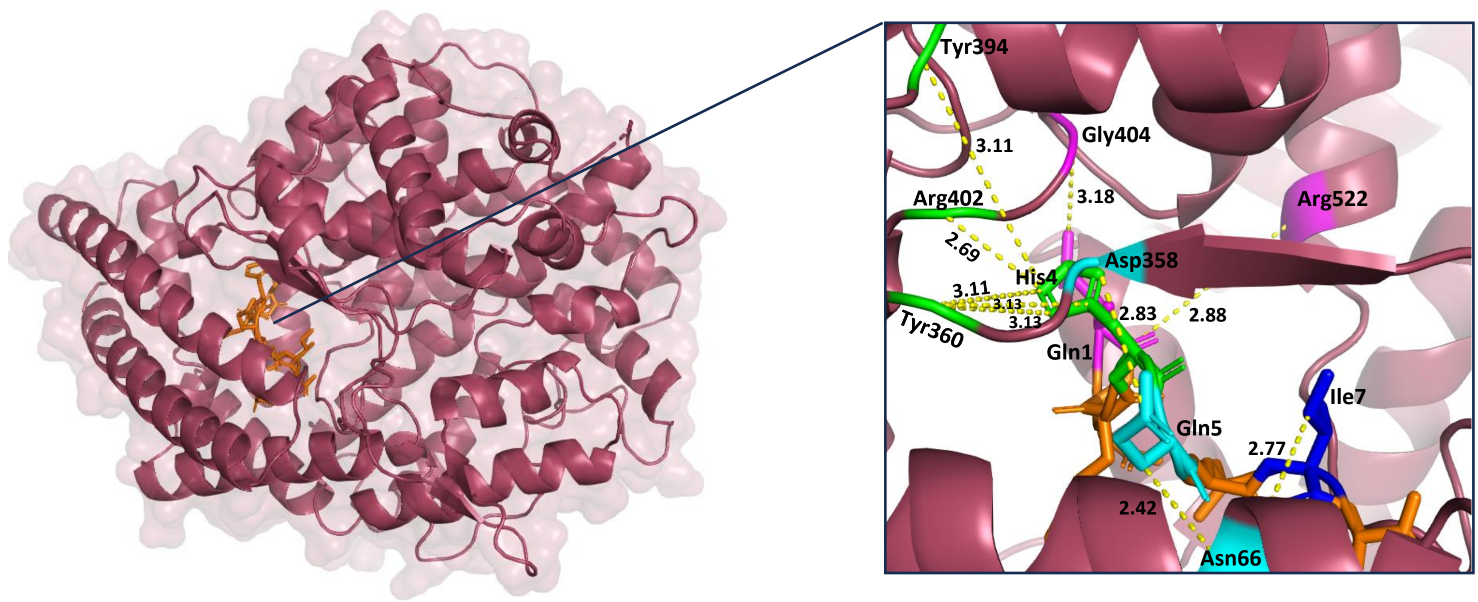

j) QDQHQKIR with ACE-I

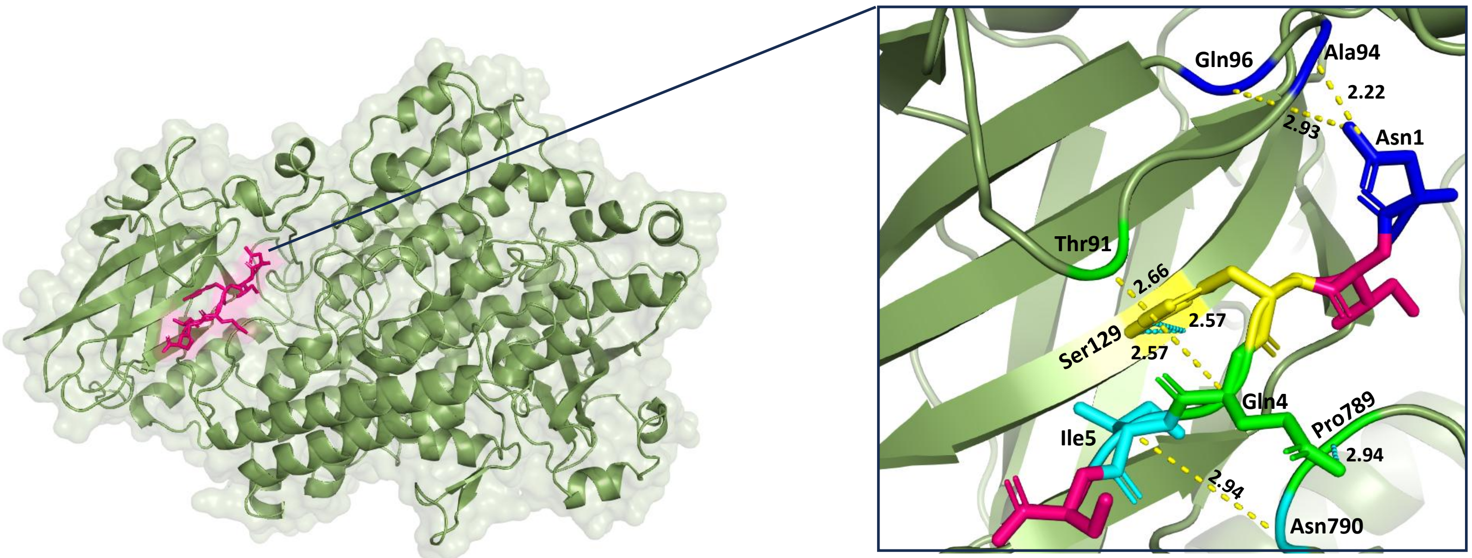

k) NIYQIS with Lipoxygenase

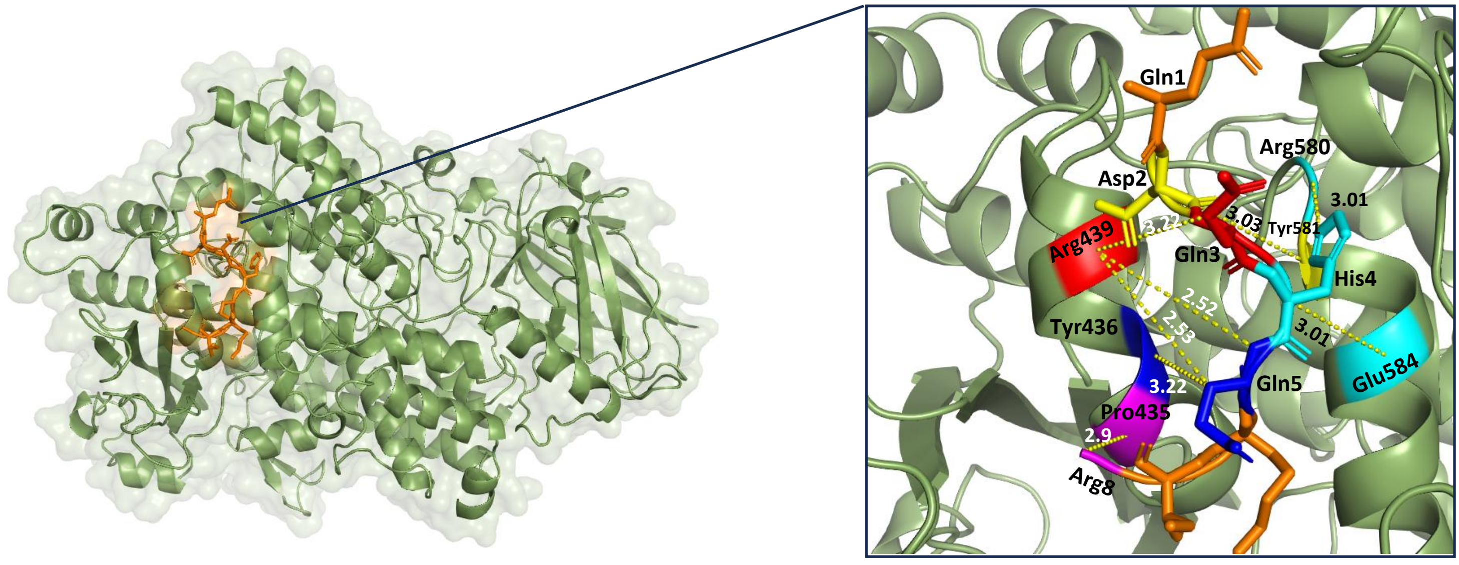

l) QDQHQKIR with Lipoxygenase

Figure S3. Mass spectrum analysis of quinoa-derived peptides (from simulated hydrolysis with stem bromelain) YDDER, NIYQIS, and QDQHQKIR after being chemically synthesized.

YDDER

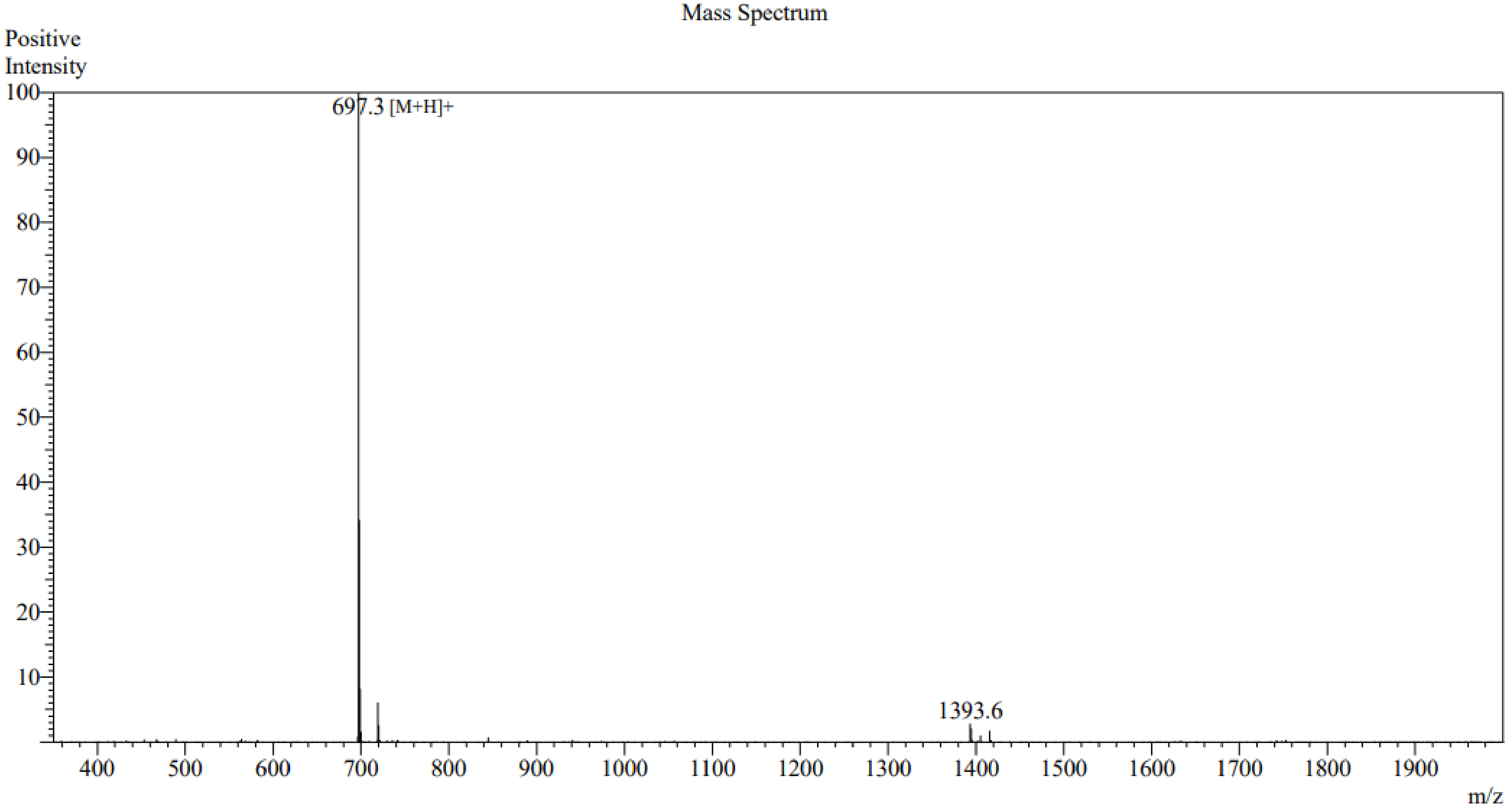

Sample Information
Month-Day Processed : 02/16/24
Time Processed : 18:57:41
Injection Volume : 0.3
Sample Name : Peptide 5
Sample ID : U183RJGNG0-9
Theoretical MW : 696.67
Observed MW : 696.3

Interface :ESI
Nebulizing Gas Flow :1.5L/min
CDL Temp :250
Block Temp :200

Equipment : ZJ21010035
Interface Bias : +4.5 kV
Drying Gas Flow :5 L/min
T.Flow :0.2 ml/min
B.conc :50%H2O/50%MeOH

NIYQIS

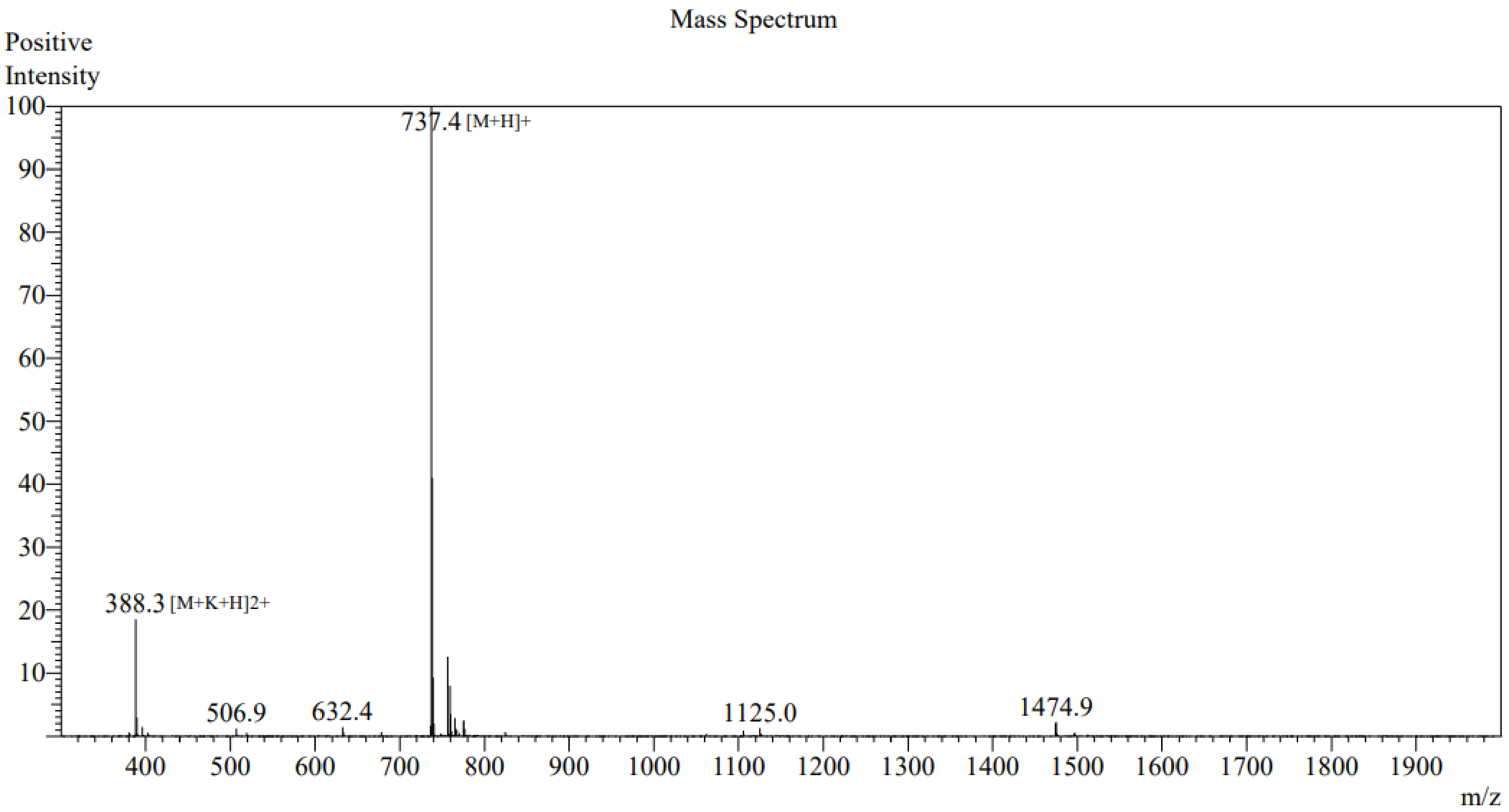

Sample Information
Month-Day Processed : 02/18/24
Time Processed : 12:38:15
Injection Volume : 0.4
Sample Name : Peptide 6
Sample ID : U183RJGNG0-11
Theoretical MW : 736.82
Observed MW : 736.4

Interface :ESI
Nebulizing Gas Flow :1.5L/min
CDL Temp :250
Block Temp :200

Equipment : ZJ21010035
Interface Bias : +4.5 kV
Drying Gas Flow :5 L/min
T.Flow :0.2 ml/min
B.conc :50%H2O/50%MeOH

QDQHQKIR

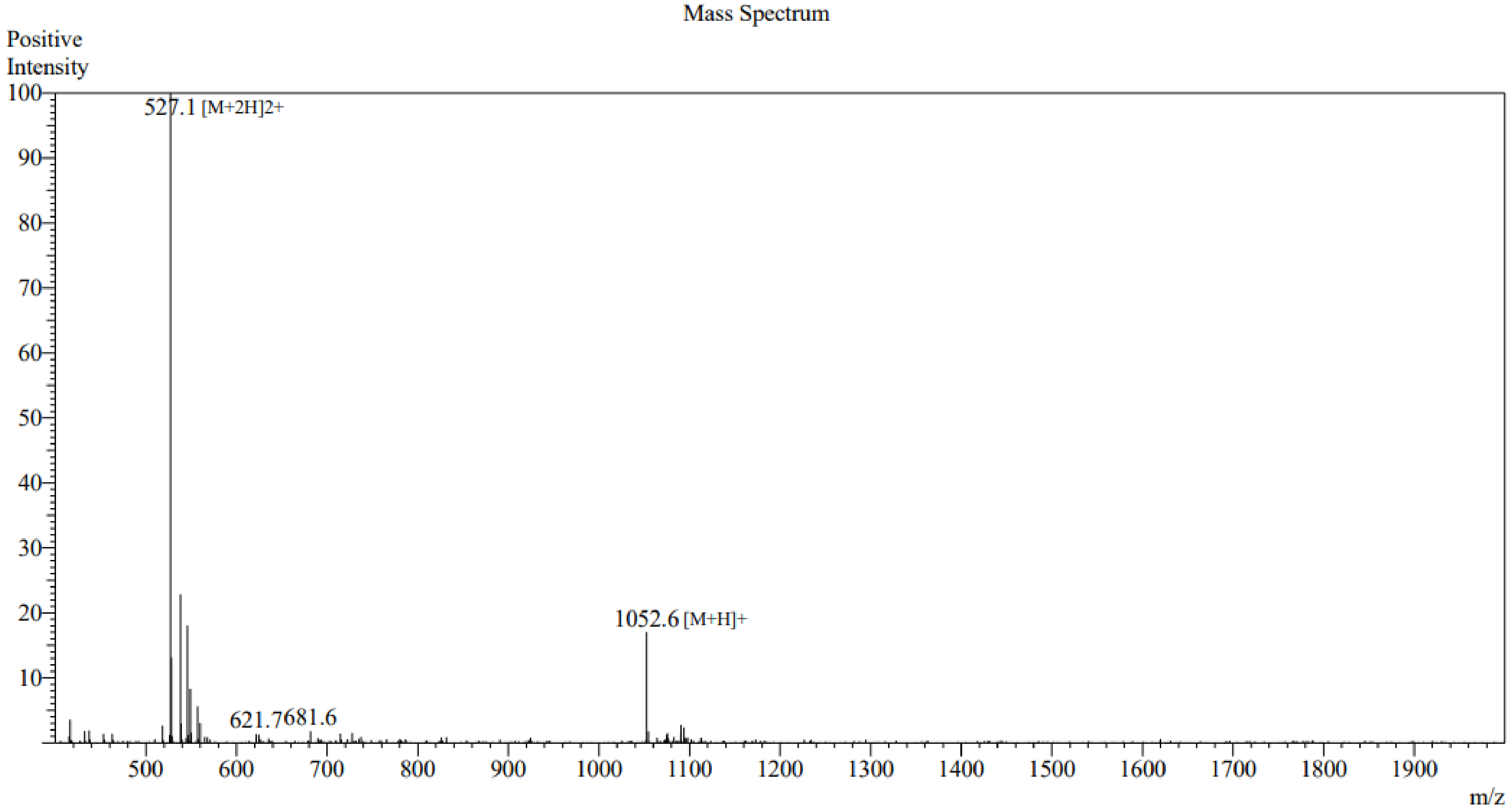

Sample Information
Month-Day Processed : 02/17/24
Time Processed : 12:11:11
Injection Volume : 0.3
Sample Name : Peptide 4
Sample ID : U183RJGNG0-7
Theoretical MW : 1052.15
Observed MW : 1052.2

Interface :ESI
Nebulizing Gas Flow :1.5L/min
CDL Temp :250
Block Temp :200

Equipment : ZJ21010035
Interface Bias : +4.5 kV
Drying Gas Flow :5 L/min
T.Flow :0.2 ml/min
B.conc :50%H2O/50%MeOH
